# Tracking Progress Toward Urban Nature Targets Using Landcover and Vegetation Indices: A Global Study for the 96 C40 Cities

**DOI:** 10.1029/2023GH000996

**Published:** 2024-02-26

**Authors:** Greta K. Martin, Katelyn O’Dell, Patrick L. Kinney, Marcia Pescador‐Jimenez, David Rojas‐Rueda, Robert Canales, Susan C. Anenberg

**Affiliations:** ^1^ The George Washington University Milken Institute of Public Health Washington DC USA; ^2^ Boston University School of Public Health Boston MA USA; ^3^ Department of Environmental and Radiological Health Sciences Colorado State University Fort Collins CO USA; ^4^ Colorado School of Public Health Colorado State University Fort Collins CO USA

**Keywords:** 0230 impacts of climate change: human health, 1640 remote sensing

## Abstract

Access to urban natural space, including blue and greenspace, is associated with improved health. In 2021, the C40 Cities Climate Leadership Group set 2030 Urban Nature Declaration (UND) targets: “Quality Total Cover” (30% green area within each city) and “Equitable Spatial Distribution” (70% of the population living close to natural space). We evaluate progress toward these targets in the 96 C40 cities using globally available, high‐resolution data sets for landcover and normalized difference vegetation index (NDVI). We use the European Space Agency (ESA)'s WorldCover data set to define greenspace with discrete landcover categories and ESA's Sentinel‐2A to calculate NDVI, adding the “open water” landcover category to characterize total natural space. We compare 2020 levels of urban green and natural space to the two UND targets and predict the city‐specific NDVI level consistent with the UND targets using linear regressions. The 96‐city mean NDVI was 0.538 (range: 0.148, 0.739). Most (80%) cities meet the Quality Total Cover target, and nearly half (47%) meet the Equitable Spatial Distribution target. Landcover‐measured greenspace and total natural space were strong (mean *R* ^2^ = 0.826) and moderate (mean *R* ^2^ = 0.597) predictors of NDVI and our NDVI‐based natural space proximity measure, respectively. The 96‐city mean predicted NDVI value of meeting the UND targets was 0.478 (range: 0.352–0.565) for Quality Total Cover and 0.660 (range: 0.498–0.767) for Equitable Spatial Distribution. Our translation of the area‐ and access‐based metrics common in urban natural space targets into the NDVI metric used in epidemiology allows for quantifying the health benefits of achieving such targets.

## Key Points

- C40 cities vary greatly in their type, extent, and distribution of natural space, including both green and blue spaces
- Roughly 80% of C40 cities meet at least one Urban Nature Declaration (UND) target, while almost half meet both goals
- We converted UND targets into the Normalized Difference Vegetation Index scale for future health impact assessments

## 1 Introduction

Urban greenspace (e.g., parks, tree‐lined streets) is associated with health benefits, operating through pathways that include increased physical activity, social interaction, sunlight and microorganism exposure, and reduced heat, air pollution, and noise exposure (de Keijzer et al., 2019; Garrett et al., 2020; Gascon et al., 2018; Nieuwenhuijsen et al., 2018; Rojas‐Rueda et al., 2019; Schinasi et al., 2019; Twohig‐Bennett & Jones, 2018; Yang et al., 2021). Urban blue space, defined as all visible surface water, may also provide similar health benefits, though the evidence is less established (Georgiou et al., 2021).

Several organizations have published guidelines for expanding and enhancing urban nature to reduce climate risk and vulnerability while improving overall health and well‐being. The World Health Organization (WHO) recommends a minimum of 0.5 ha (5,000 square meters) of public greenspace within 300 m of a person's home (*Urban Green Spaces: A Brief for Action*, 2017). With 31 city signatories, C40 cities, an international network of mayors committed to reducing greenhouse gas emissions, established an Urban Nature Declaration (UND) that included the following two 2030 targets: (a) Quality Total Cover: “30%–40% of total built‐up city surface area will consist of green spaces… or permeable spaces”, and (b) Equitable Spatial Distribution: “70% of city population has access to green or blue public spaces within a 15‐min walk or bike ride” (C40 cities, 2021). Some cities have also made individual commitments to expanding urban nature. Within the C40 network, for example, Philadelphia, USA, has set a goal of achieving 30% tree canopy cover by 2025 (Kondo et al., 2020); London, England, has pledged to become the first “national park city” with half of its area designated as greenspace (*London Environment Strategy*, 2018); and Medellín, Colombia launched the Green Corridors project from 2016 to 2019, which planted trees along 20 km of roads and waterways (C40 Cities Climate Leadership Group, Nordic Sustainability, 2019).

Urban goals for expanding nature often have multiple objectives, including mitigating greenhouse gases, enhancing urban resilience to climate‐sensitive hazards, and promoting healthier communities. Tracking progress toward these goals, and in particular understanding the health benefits from achieving them, could provide critical information to mayors, urban networks such as C40, civil society, and the public more broadly. Quantifying the health benefits of urban nature goals is critical because such gains are more immediate than those from reducing carbon emissions, from increased active transport for example, and more certain than those of resilience to extreme weather events, like flooding or heat waves. While such an assessment could help to evaluate societal improvements and make evidence‐based changes as needed, there is a disconnect between urban nature policies and the health literature. Most epidemiological studies of greenspace and health outcomes use the normalized difference vegetation index (NDVI) (S. Huang, Tang, et al., 2021). For this reason, exposure‐response functions linking greenspace to nature are generally measured using increments of NDVI (Rojas‐Rueda et al., 2019; Yuan et al., 2021). Only two studies to date have estimated health benefits of expanding green space in many cities globally; both used NDVI increments as metrics for characterizing green space (Barboza et al., 2021; Brochu et al., 2022) and one also used percent green area (Barboza et al., 2021).

NDVI is a satellite‐derived measure that uses visible and near‐infrared light to quantify vegetation density. It ranges from −1 to 1, with higher positive values indicating healthier, denser vegetation, values near 0 suggesting barren land, and negative values marking water, snow, and ice (*Measuring Vegetation (NDVI & EVI)*, 2000). The advantages of NDVI are that it can differentiate not only vegetation from built surfaces but also the health and density of vegetation. Additionally, NDVI has full global coverage with fine spatial (10 m) and temporal (10 days) resolution. NDVI also captures smaller‐scale vegetation, such as tree‐lined streets and small parks, which is important in characterizing the amount of greenspace people are exposed to in cities. Key limitations of the NDVI metric are that it does not capture the type, accessibility, or usability of greenspace, which are often considered in urban greenspace targets in practice. Furthermore, because NDVI is not an intuitive metric, decision makers generally rely on other measures of nature, making it challenging to quantify the health gains of urban nature policies.

Studies examining the health benefits of blue space have employed a wide range of metrics. For example, in a systematic review of 50 studies on the relationship between blue space and health, 17 different measures of blue space were used (Georgiou et al., 2021). Methods for assessing exposure to blue space were divided into four broad categories: measures of the amount of blue space within a given area, distance to blue space, contact with blue space, and visibility of blue space (Georgiou et al., 2021). The most common categories used in the epidemiological literature were measures of the amount of blue space within a geographical area and the distance to blue space. However, there is substantial variation within these categories. For example, studies considering the amount of blue space within a given area used buffers ranging in size from 100 m to 1.5 km and, in some cases, relied on administrative zones such as zip codes (Georgiou et al., 2021). Due to the inconsistent measurement of blue space, there is not a commonly accepted exposure‐response function linking surface water and health outcomes.

This paper has three main objectives: (a) characterize the extent and distribution of urban green and urban green and blue combined, or natural space, in C40 cities using satellite‐based metrics; (b) evaluate progress toward C40's UND targets; and (c) convert the UND targets into a city‐specific metric that can be used with NDVI‐based epidemiological exposure‐response functions to estimate the health benefits of achieving the targets. For the third objective, we follow a similar approach to health impact assessments conducted for Philadelphia, USA (Kondo et al., 2020) and European cities (Barboza et al., 2021) to convert the Quality Total Cover target into NDVI and expand on this approach to address the Equitable Spatial Distribution target. We conducted our analysis for all 96 cities in the C40 network, accounting for 291 million residents, 1,747 megatons of greenhouse gas emissions, and a gross domestic product of nearly $11 billion (Hoornweg et al., 2020). These cities represent 48 countries across six continents. The methods we use to convert these goals to the NDVI scale could also be applied to evaluate progress toward additional policy targets aimed at expanding the amount of and access to urban nature.

## 2 Methods

**Figure 1:**
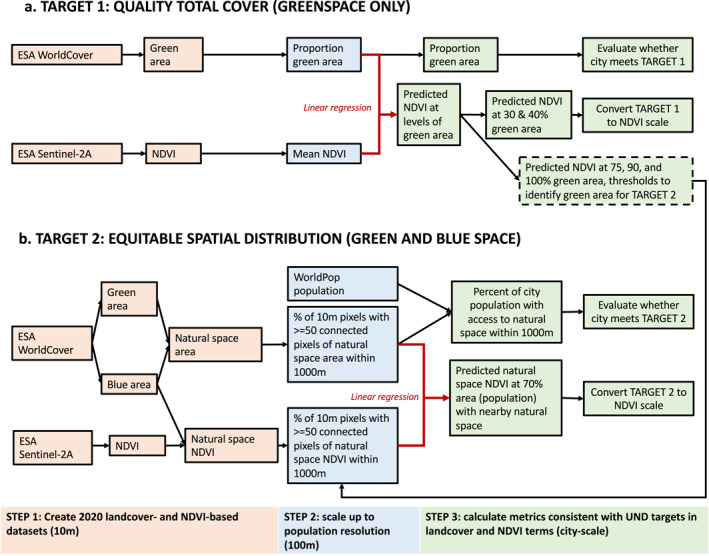
Flowchart of methods used to evaluate whether cities meet the two Urban Nature Declaration targets and to convert the targets to the normalized difference vegetation index scale. The colors indicate the analytical steps and spatial resolution of the data.

This study took a multi‐step approach to characterize and evaluate urban natural space against the UND targets and convert the UND targets into a city‐specific NDVI metric across all 96 cities of the C40 network (Figure 1). We leveraged the full geographical coverage and high spatial resolution of satellite‐derived landcover and NDVI to quantify greenspace and total natural space, inclusive of green and blue space, in each city for 2020 (Figure 1, step 1). We then scaled up these data sets to the resolution of our population data set (100 m) and ran city‐specific regression models to understand the relationship between the landcover‐ and NDVI‐based metrics (Figure 1, step 2). Finally, we used the landcover data sets to evaluate each city's current extent and distribution of natural space against both UND targets and estimate the equivalent level of natural space needed to meet each target on the NDVI scale (Figure 1, step 3). For Quality Total Cover we used greenspace only (Figure 1a) and for Equitable Spatial Distribution we used total natural space (Figure 1b), aligned with the quantities used in the targets. The data inputs, in map format, are shown in the Supporting Information S1 for an example city, Washington, DC (Figure S1 in Supporting Information S1).

### 2.1 Characterizing Urban Natural Space

To characterize natural space for each city, we used two global, 10 m × 10 m gridded data sets for the year 2020: (a) the European Space Agency's (ESA) Copernicus Sentinel‐2A satellite images (ESA, 2020) to calculate NDVI, and (b) land classifications from the ESA's WorldCover data set (Zanaga et al., 2021).

#### 2.1.1 Defining Greenspace

To estimate greenspace extent from ESA Sentinel‐2A, we first calculated NDVI using the near‐infrared (“B8”) and visible light (“B4”) bands (Equation 1; Rouse et al., 1974).

(1) $$\text{NDVI}=\frac{\left(\text{NIR}-\text{VIS}\right)}{\left(\text{NIR}+\text{VIS}\right)},$$

where NIR is near‐infrared, and VIS is visible light. Following previous studies (Corbane et al., 2020; C. Huang, Tang, et al., 2021; Lindsay et al., 2022; Pericak et al., 2018; Sonia et al., 2022; You et al., 2021), we then selected the day with the greenest value (highest NDVI) from all the 2020 images for each pixel to eliminate cloudy pixels and capture the greenest season across cities in the Northern and Southern hemispheres. This choice captures peak greenness in each city, which may overestimate the average conditions. However, any bias should be non‐differential across cities and consistent in both our estimates of actual and target NDVI levels.

We separately created a binary definition of greenspace, mirroring the Quality Total Cover target language. We included seven of the 11 land cover classifications in the 2020 ESA WorldCover data set: trees, shrubland, grassland, cropland, herbaceous wetland, mangroves, and moss and lichen. We excluded the other four categories which were not indicative of vegetation: built‐up, barren/sparse vegetation, snow and ice, and open water. WorldCover is an independently validated global data set with an overall accuracy of 74.4% (Zanaga et al., 2021).

#### 2.1.2 Defining Natural Space

We defined natural space as any green or blue space. While other natural landscapes exist, such as rock and snow, we consider only green and blue spaces, as these are the types of nature included in the UND targets and whose health benefits are best supported by the literature. In both our NDVI‐ and landcover‐based definitions of natural space, we used the ESA WorldCover classification of “open water” to identify surface water at the 10 m pixel level. We combined the landcover water classification with NDVI by assigning water pixels a value of 1, equating blue space with the highest possible NDVI value. In the rare case (*N* = 204, <0.0001%) where pixels were not identified as water by the landcover data set but had a negative NDVI value indicative of clouds or water, they were also considered blue spaces. For the landcover‐based definition of natural space, we included any open water pixel in the binary classification.

### 2.2 Evaluating Performance Against UND Targets

We used the landcover‐based greenspace and natural space data sets to compare 2020 levels of urban natural space to the Quality Total Cover and Equitable Spatial Distribution targets, as these definitions align best with the UND target definitions of nature.

#### 2.2.1 Evaluating Progress Toward Quality Total Cover Using Greenspace

We used our landcover definition of greenspace to evaluate urban performance against the Quality Total Cover target, which does not include blue space. While the language of the UND target allows for “permeable surfaces” as well as greenspace, we have only included greenspace in our definition. We aggregated this binary data set, where each native 10 m pixel was classified as greenspace or not, to the 100 m resolution by taking the area‐weighted mean, with each new 100 m pixel representing the percentage of 10 m pixels that were classified as green area (Figure S1a in Supporting Information S1). Though the population distribution is not relevant for this target, we first aggregated to the 100 m resolution for efficiency and to harmonize the data processing steps with those of the Equitable Spatial Distribution target which does incorporate population data. We then took the mean of all 100 m pixels within each urban area to evaluate the city‐wide proportion of green area.

#### 2.2.2 Evaluating Progress Toward Equitable Spatial Distribution Using Natural Space

We used the natural space data set to evaluate performance against the Equitable Spatial Distribution target, which considers the proximity of the population to both green and blue space. We first identified areas with sizable, contiguous natural space extents for each city to exclude most private lawns and gardens since this target calls for population proximity to *public* green or blue space. Without another source to derive the minimum natural space area that can reasonably be considered public, we used a threshold value of 0.5 ha (5,000 m^2^), used in the WHO definition of universal access to greenspace (*Urban Green Spaces: A Brief for Action*, 2017). We then created 1,000 m buffers around each 10 m native pixel and flagged whether there was at least 0.5 ha of natural space in that zone to capture population access within a fifteen‐minute walk or bike, as specified by the Equitable Spatial Distribution target. We chose this distance based on The Federal Highway Administration guideline that the average person can walk 1,080 m in 15 minutes (Turner et al., 2006). While the average cyclist can travel farther, we chose to focus solely on walking for a more inclusive definition of access, as cities vary greatly in cycling infrastructure, bike ownership, and bike comfortability. Next, we aggregated this data set to the 100 m resolution, using the area‐weighted mean. The result was a 100 m resolution data set where each grid cell represents the percentage of an area within that pixel with access to 0.5 ha or more of natural space within a 1,000 m buffer or fifteen‐minute walk (Figure S1c in Supporting Information S1). In the final step, because this target is dependent on the spatial distribution of the population, we multiplied the green and blue landcover data by the population living in the corresponding grid cell to determine the proportion of the population across the city with proximity to natural space.

### 2.3 Converting UND Targets to the NDVI Scale

We next converted the natural space targets into a city‐specific NDVI metric that can be used with NDVI‐based epidemiological exposure‐response functions to estimate the health benefits of achieving the UND targets.

#### 2.3.1 Converting Quality Total Cover Target to NDVI

For the Quality Total Cover target, which focuses on greenspace, we fit ordinary least squares models, regressing the proportion of green area from Section 2.2.1. on the corresponding mean NDVI value for each 100 m grid cell, following methods used in a health impact assessment of Philadelphia's tree canopy goals (Kondo et al., 2020). We fit separate regression models for each of the 96 cities to account for differences in local climate and greenness. Finally, we used these models to predict the NDVI value associated with 30% and 40% green area in each city, corresponding to the minimum target range for the Quality Total Cover target. We assessed model fit using the coefficient of determination (*R* ^2^) and the root mean square error (RMSE).

#### 2.3.2 Converting Equitable Spatial Distribution Target to the NDVI Scale

To convert the Equitable Spatial Distribution target to NDVI terms, we first set a threshold NDVI value above which a 10 m pixel would be considered “green.” Using the regression models from Section 2.3.1., we predicted the NDVI value associated with 75%, 90%, and 100% green area, which we then used as thresholds to determine natural space pixels in our natural space NDVI data set. Because water pixels were assigned a value of one in this data set, water pixels were always included as natural space, regardless of the chosen threshold. Next, we paralleled the process used for the landcover data set, flagging 10 m pixels with natural space areas of 0.5 ha or more within a 1,000 m buffer. We then aggregated this binary data set to the 100 m resolution using an area‐weighted mean. Finally, we regressed the landcover‐derived proportion of area with access to at least 0.5 ha of contiguous natural space within a 1,000 m buffer on the NDVI‐based equivalent data set (Figure S1d in Supporting Information S1). We assessed model fit using the coefficient of determination (*R* ^2^) and the RMSE.

### 2.4 Characterizing Urban Population and Spatial Extent

As the Equitable Spatial Distribution target relates the proximity of natural space to the urban population, we assessed the co‐location of natural space and population for this target. We used 100 m gridded world population estimates for 2020 from WorldPop (Bondarenk et al., 2020). We included only the population aged 20 years and older, as meta‐analyses linking greenspace and all‐cause mortality have been limited to adult populations.

We defined the spatial bounds of each city using the Global Human Settlement Urban Center database (Global Human Settlement Urban Centre database (GHS‐UCDB)) (European Commission Joint Research Centre, 2019). The GHS‐UCDB uses population data and built‐up surface area to define city bounds corresponding to where concentrated populations live rather than administrative bounds. We chose this urban extent definition because it provides a standardized boundary methodology across our diverse city population. We conducted a sensitivity analysis using self‐defined urban bounds from C40 cities (Figure S2 in Supporting Information S1) to evaluate how the definition of the urban area impacts estimated natural space extents and urban nature targets (Datasets S1 and S2 in Supporting Information S1).

## 3 Results

### 3.1 Extent of Natural Space Across C40 Cities

**Figure 2:**
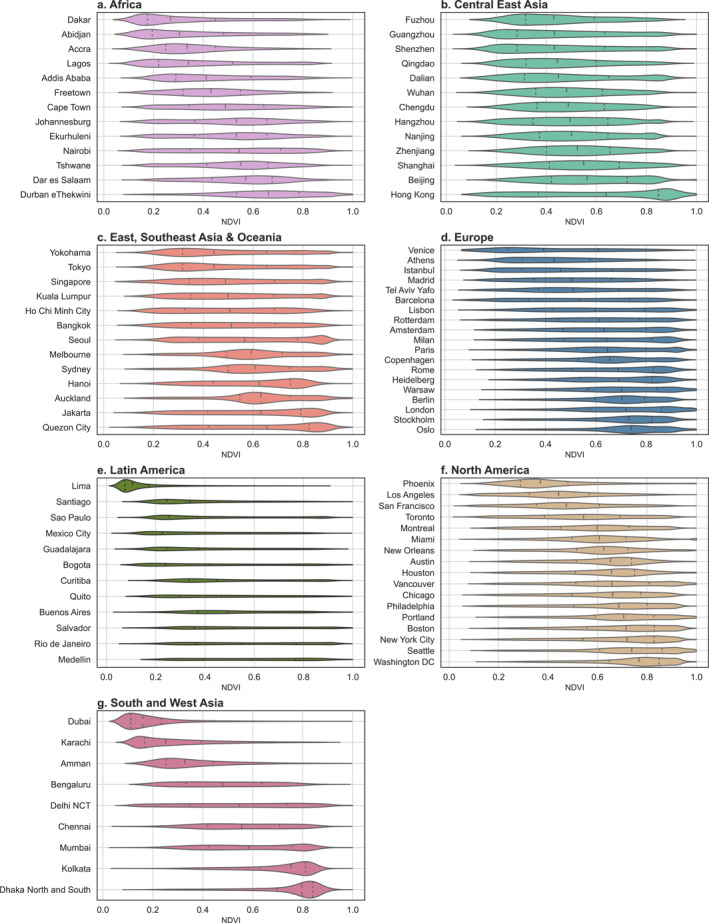
Distribution of maximum 2020 normalized difference vegetation index (NDVI) values for each 100 m pixel in C40 cities within each world region. Quartiles of NDVI are indicated by dashed vertical lines. These distributions do not include blue space.

Cities vary greatly in their extent and distribution of greenspace (Figure 2, Figures S3–S5 in Supporting Information S1). The overall city mean NDVI across C40 cities was 0.538 and ranged from 0.148 in Lima, Peru, to 0.739 in Dhaka, Bangladesh (Dataset S1 in Supporting Information S1). Even for cities with similar median NDVI values, their distribution of greenspace can differ dramatically. For example, Hanoi, Vietnam; Auckland, New Zealand; and Jakarta, Indonesia, have a median NDVI of approximately 0.62, while their distribution of grid cell values is very different (Figure 2c). European and North American cities tended to have higher median NDVI values, and Latin American cities tended to have lower ones. However, the intra‐regional variability was more substantial than regional differences. The extent of natural space increased in most cities when considering the natural space NDVI data set, which includes blue space (Figure S4 in Supporting Information S1). The overall city mean natural space NDVI was 0.569 (range: 0.181–0.816). Adding blue space changed city‐mean NDVI the most in Venice, Italy, where the inclusion of water resulted in a natural space NDVI that was 87% greater than its greenspace‐only NDVI value. Dakar, Senegal, and Dubai, United Arab Emirates, also gained substantial natural space with the inclusion of water, with natural space NDVI values increasing by over 40%. Despite this overall trend, there were six C40 cities whose NDVI value increased by less than 0.1% when blue space was considered: Addis Ababa, Ethiopia; Quito, Ecuador; Amman, Jordan; Tshwane, South Africa; Guadalajara, Mexico; and Nairobi, Kenya (Dataset S1 in Supporting Information S1).

The city mean proportion of green urban area in the landcover‐based data set was 0.427. Compared with using NDVI, measuring greenspace using the landcover data set resulted in more extreme values, ranging from a city‐mean of 0.031 in Lima, Peru, to 0.806 in Dhaka, Bangladesh. Despite averaging the 10 m native pixels to the 100 m resolution in this data set, the distribution of pixel values remained highly clustered near 0 and 1 (Figure S3 in Supporting Information S1). The relative order of greenness between cities remained fairly consistent between the greenspace and NDVI metrics (Figure S3 in Supporting Information S1 & Figure 2). Adding blue space to this measure increased the mean proportion of green or blue urban areas to 0.464 (range: 0.068–0.816). Including water in the landcover‐based data set had a more dramatic effect than on NDVI. The addition of blue space increased the natural space value by almost 300% in Dubai, United Arab Emirates, nearly tripled it in Venice, Italy, and more than doubled in Lima, Peru. The same cities that were largely unchanged by adding water to the NDVI metric saw a similarly modest increase in the landcover metric. Of this group, no city experienced a greater than 0.1% increase, except for Guadalajara, Mexico, whose value rose by 0.14%.

### 3.2 Performance on UND Targets

**Figure 3:**
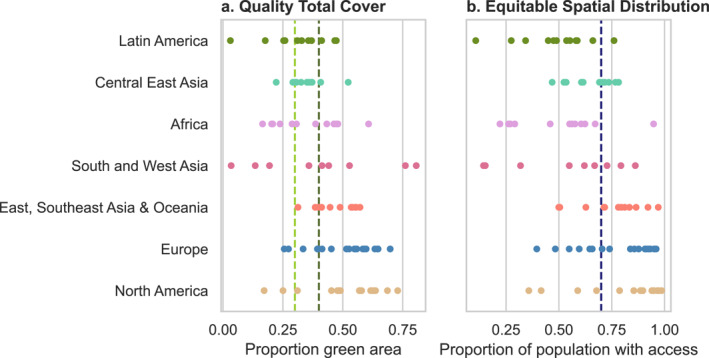
Green and natural space across C40 cities by region in 2020, quantified using metrics comparable to the Quality Total Cover (panel a) and Equitable Spatial Distribution (panel b) Urban Natural Declaration targets. The scatter points represent cities and colors correspond to the region colors in Figure 2. The vertical lines in panel a mark the Quality Total Cover minimum goal range (0.30–0.40 of the urban area is greenspace) while the vertical line in panel (b) represents the Equitable Spatial Distribution target (0.70 of population has access to blue or greenspace within a 15‐min walk).

Many C40 cities already met the standard of one or both UND targets (Figure 3). Seventy‐seven (80%) of cities met the lower end of the Quality Total Cover target, with at least 30% of their urban area designated as greenspace. At least 60% of cities in all regions met the 30% Quality Total Cover target, including all 13 cities in the East, Southeast Asia, and Oceania region (Figure 3). Nearly 90% of North American and European cities met the higher end of this target range, with 40% or more greenspace. Despite these regional trends, there was substantial intra‐regional variation in performance on the Quality Total Cover target.

Fewer cities met the Equitable Spatial Distribution target; 70% of the population has access to green or blue space within a 15‐min walk in 45 C40 cities. There was considerable inter‐ and intra‐regional variation on this target. Over 75% of North American C40 cities met the Equitable Spatial Distribution target, compared to less than 10% of C40 cities in the Latin American and African regions. Less than 20% of the population has access to natural space within a 15‐min walk in Lima, Peru; Karachi, Pakistan; and Dubai, United Arab Emirates. In contrast, there are 18 C40 cities, representing four of the seven regions, with over 90% of the population having nearby natural space. All cities that met the Equitable Spatial Distribution target also met the Quality Total Cover target, resulting in 45 cities that met both UND targets.

### 3.3 Converting UND Targets to the NDVI Scale

**Figure 4:**
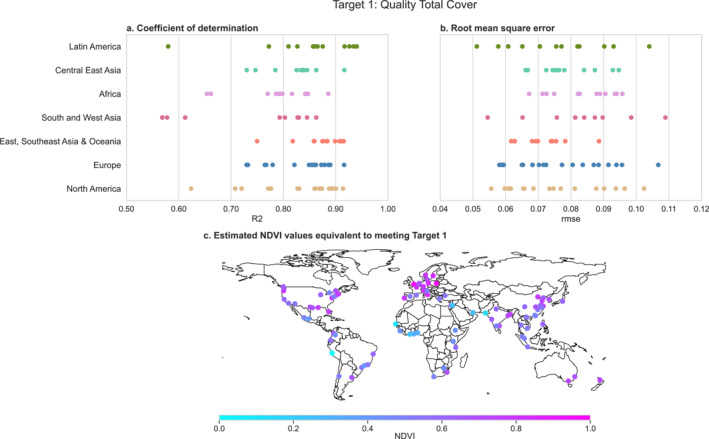
Fit statistics and predicted normalized difference vegetation index (NDVI) values for the regression models used to convert the Quality Total Cover target to the NDVI scale. Each dot represents a city. Panels (a) and (b) show the model adjusted *R* ^2^ and root mean square error by region, respectively. Panel (c) shows the predicted NDVI value where the proportion of green area is 0.3, aligned with the lower minimum threshold proportion of greenspace in the Quality Total Cover target.

After comparing each city's existing levels of natural space to the UND targets using landcover data sets, we translated these targets into the NDVI scale so that the health benefits of meeting the UND targets may be quantified using NDVI‐based exposure‐response functions. For the Quality Total Cover target, we modeled the relationship between the proportion of green area and NDVI in each 100 m pixel by running separate linear regression models for each city. These models generally fit well (Figures 4a and 4b). On average, the models explained 83% of the variance in NDVI, ranging from 57% to 94% for individual cities. The RMSE for these models had a mean of 0.077 (range: 0.051, 0.101) across C40 cities. For an average city and pixel, predicted NDVI values differed from the actual NDVI values by 0.077. In general, the Quality Total Cover regressions had better fit in cities with more greenspace (Figures S6–S12 in Supporting Information S1). We used our models to predict the NDVI value equivalent to achieving the Quality Total Cover target for each city. The mean NDVI representing 30% green area was 0.478 (range: 0.352, 0.565) across all cities (Figure 4c). At 40% green area, the mean predicted NDVI was 0.528 (range: 0.428, 0.612). In our sensitivity analysis, using the C40 urban boundaries had little effect on our estimates of the NDVI‐equivalent level of the Quality Total Cover target (Figure S13a in Supporting Information S1).

We also used the regression models to predict threshold NDVI values at or above which a pixel would be classified as “green” to quantify the Equitable Spatial Distribution target in NDVI terms. We tested three thresholds: the predicted NDVI value where the percent of green area was 75%, 90%, and 100%. We selected the NDVI prediction at 75% green area to classify pixels as greenspace, because the fit statistics for the Equitable Spatial Distribution regressions performed best with this threshold. The fit statistics and model predictions using 90% and 100% proportion green area can be found in the Supplemental Information (Figures S14 and S15 in Supporting Information S1).

**Figure 5:**
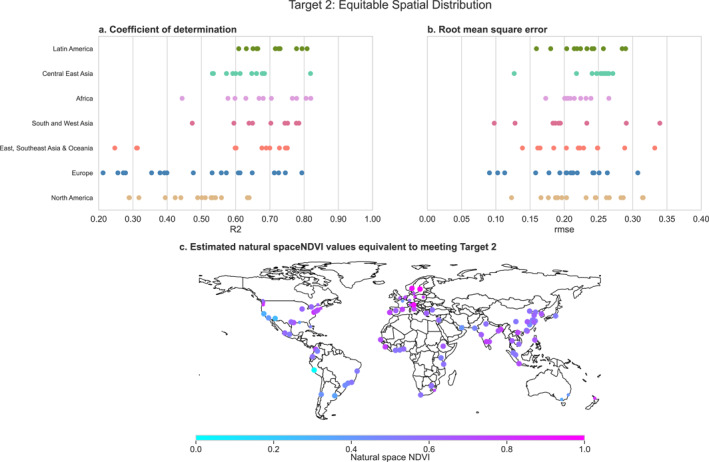
Fit statistics and predicted normalized difference vegetation index (NDVI) for the regression models used to convert the Equitable Spatial Distribution target to the NDVI scale. Each dot represents a city. Panels (a) and (b) show the model fit statistics by region. Panel a shows the adjusted *R* ^2^ value, while Panel (b) shows the root mean square error. Panel (c) shows the predicted natural space NDVI value where 0.70 of the area, and thus population, has access to sufficient nearby natural space, aligned with the Equitable Spatial Distribution target. Models with poor fit (*R* ^2^ less than 0.50) are shown with smaller dots.

We used linear regression models to translate our landcover definition of the Equitable Spatial Distribution target to the NDVI scale. These models had a mean *R* ^2^ across cities of 0.597 (range: 0.213, 0.820) and a mean RMSE of 0.221 (range: 0.091, 0.340) (Figures 5a and 5b). The Equitable Spatial Distribution regressions tended to fit best when the proportion of the population with nearby natural space was less than 90% (Figures S16–S22). We used these regressions to predict the natural space NDVI value equivalent to achieving the Equitable Spatial Distribution target of 70% population access to natural space with a 1,000 m buffer or 15‐min walk. The average natural space NDVI associated with meeting this UND target was 0.660, ranging from 0.498 to 0.767 across C40 cities (Figure 5c). In our sensitivity analysis using C40 urban boundary definitions, we found that the predicted natural space NDVI value equivalent to meeting the Equitable Spatial Distribution target was generally higher in whichever urban boundary definition was larger (Figure S13b in Supporting Information S1).

## 4 Discussion

In this assessment of urban greenspace and natural space across 96 global cities, we found that C40 cities vary greatly in their amount, type, and distribution of natural spaces. While much of the literature on urban nature has focused solely on greenspace, our results show that blue space can greatly contribute to urban natural space in many cities. For some cities, including water in the definition of natural space made a substantial impact, in some cases doubling the estimated amount of natural space within city bounds. We compared existing levels of urban natural space to the C40 UND targets and found that most C40 cities already meet one or both targets. Of the 96 C40 cities, 77 (80%) have at least 30% green area (Quality Total Cover target), while at least 70% of the population has access to green or blue space within a 15‐min walk in 45 (47%) cities (Equitable Spatial Distribution target). Finally, we converted the C40 policy targets to the NDVI scale, making our natural space exposure assessment interoperable with exposure‐response functions found in the health literature. The city‐specific equivalent NDVI value to meet the Quality Total Cover target ranged from 0.352 to 0.565, and the natural space NDVI value for the Equitable Spatial Distribution target ranged from 0.498 to 0.767. These translations can be used to quantify the health gains from expanding urban nature.

Our work builds on a body of research to both quantify urban exposure to greenspace across global cities and estimate its health implications. In terms of exposure assessment, our city‐wide estimates of NDVI were consistently higher than the 1 km population‐weighted peak (greenest day) NDVI values reported for 2020 in a recent study of 1,000 global cities (Stowell et al., 2023), with a mean difference of 0.19 and a standard deviation of 0.05). However, our estimates had a strong correlation of 0.91 with the Stowell et al. measure, despite the difference in resolution and population weights. This difference is in part due to our decision to use the greenest pixel from 2020 to measure greenspace, as our study population of cities have very different seasons. While this choice likely exaggerates the greenness of a city, it should be non‐differential across cities. Furthermore, both our estimates of the actual and target NDVI will be biased in the same direction and magnitude by this decision, which should limit the systematic error in future calculations of the gap between the current and ideal natural space levels needed for health impact assessments. We assessed natural space at a finer scale (10 m) than most health and exposure studies, which commonly use satellite images from the Landsat (30 m) or Modis (100 m) satellites (S. Huang, Tang, et al., 2021). This is important for capturing urban greenspace, which often consists of smaller spaces.

Health impacts assessments to date have focused on American and European cities and considered only greenspace. For example, a study of populous US cities found that between 34,000 and 38,000 all‐cause deaths could have been avoided in 2000, 2010, and 2019 with an increase in NDVI of 0.1 (Brochu et al., 2022). In three additional health impact assessments, urban nature goals were used to provide more context and real‐world application. A study of European cities reported that 42,968 (95% CI 32,296–64,177) deaths could be avoided annually if the WHO universal access to greenspace target were met (Barboza et al., 2021), while an analysis of Philadelphia, USA found that 403 (95% CI 298–618) deaths could be prevented if the city were to meet its 2025 goal of 30% tree canopy cover (Kondo et al., 2020), and an investigation of Phoenix and Denver, USA found that 200 (95% CI 100–306) and 368 (95% CI 181, 558) deaths could be averted if Denver and Phoenix were to meet their urban tree canopy goals of 20% and 25% respectively (Dean et al., 2024). In this work we develop a framework for converting area‐ and access‐based measures into NDVI terms and propose one method for incorporating blue space into urban nature definitions. The methodology we follow here can be used to convert policy goals beyond the UND targets into NDVI equivalents, so that the health benefits of such actions can be estimated.

While a translation between the C40 targets and NDVI is needed to assess the health benefits of these goals using NDVI‐based exposure‐response functions, the NDVI metric is not without its limitations. First, NDVI relies solely on the greenness of an area, meaning it has no insight into the accessibility or quality of that space, which is relevant for health benefits. Public parks and private golf courses are not differentiated by the satellite. That said, some evidence suggests that even viewing green and blue spaces can have positive health benefits, such as reducing stress and anxiety and increasing productivity (Kaplan, 1993; Stephen Kaplan & Rachel Kaplan, 1989). Second, there may be forms of nature that, though neither blue nor green, present many of the same benefits as greenspace. For example, desert climates might feature sandy or rocky terrain that can be used for exercise, provide a place to gather with friends and family, and offer natural beauty. A 2022 review of natural spaces outside the “green” and “blue” paradigm looked at landscapes dominated by snow and ice, deserts, and caves and found some evidence that there are health benefits from these environments, which are not well‐represented by NDVI (Li et al., 2023). Lastly, NDVI is limited in its ability to differentiate the density of vegetation at very high levels of leaf area index (Wang et al., 2016). This systematic error could affect the accuracy of our NDVI and NDVI‐scale predictions for areas with high vegetation density. Because the UND target thresholds are not at the extremes of leaf density, this should not have a substantial effect on our results. While NDVI is imperfect, it is widely used in health and environmental literature and is easily calculated from publicly available data with full global coverage at a fine spatial and temporal scale, making it a convenient metric for international policy applications.

Beyond NDVI as a metric, there are limitations in our construction of ideal levels of urban natural space. While using the targets set by the C40 cities themselves is valuable for political buy‐in, there are some concerns about their appropriateness for such a geographically diverse group of cities. For some, achieving 30%–40% green urban area may not be the most sustainable or feasible standard. For cities with desert climates, such as Phoenix or Dubai, maintaining a 30% green area would require high water usage that could damage the environment and health or be unattainable. There is some evidence that trees have stronger health associations than grass (Reid et al., 2017). While tree canopy would have a higher NDVI value than parks, vegetation type is not differentiated in the 30%–40% greenspace target. We have included all forms of vegetation in our binary definition of greenspace representing the Quality Total Cover target including cropland, whose association with health outcomes has not been studied in detail. Additionally, policies to increase greenspace often do so where land is cheapest, leading to “green gentrification” or increased property values where new parks and greenways are added (Wolch et al., 2014). Further, the Equitable Spatial Distribution target does not capture who has access to urban nature; the 70% that have access may or may not fairly represent the larger population. We chose a 1,000 m buffer to approximate a 15‐min walk for this target. This area‐based approach may not reflect individual behaviors or outcomes due to the Uncertain Geographic Context Problem (Kwan, 2012) or capture attributes that impede or facilitate mobility (e.g., the absence or existence of sidewalks, streetlights, and other walking infrastructure). Furthermore, our definition of natural space is not limited to publicly available spaces as stipulated in the Equitable Spatial Distribution target. We compared our Equitable Spatial Distribution estimates to Trust for Public Land's ParkServe® database (Trust for Public Land, 2024), which includes a measure of the proportion of the population with access to publicly available greenspace within a 10 min or half mile (∼800 m) walk and found that for 11 of the 14 American cities for which this data is available, the proportion of the population with access was larger using our results. This aligns with the larger area used and the inclusion of water in our definition. The three cities for which the Parkserve measure was larger (Los Angeles, New York City, and San Francisco) could be a result of the mismatch in definition of urban areas, as we used the UCDB and Parkserve uses U.S. Census Urban Areas, which may have had a larger impact on these large cities. Finally, existing methods for combining green and blue space are limited (Mizen et al., 2019). In this paper, we have developed a natural space NDVI metric to allow for the inclusion of water by assigning the highest value of NDVI, 1. While evidence suggests that exposure to blue space provides similar benefits to that of greenspace, the relative strength of this relationship is unknown.

Our work provides a pathway to assess the health benefits of urban nature policies, though further work is needed in a few key areas. Further research to quantify the effect of urban blue space on health outcomes and innovation in jointly capturing the health impact of access to urban natural space is needed to provide more comprehensive and realistic information to urban planners and policymakers. Furthermore, additional methods for converting access‐based measures into NDVI terms would help quantify the associated health benefits of such policy aims. While we were able to achieve good predictions from most of our Equitable Spatial Distribution models, some had *R* ^2^ values under 0.5, which could affect the accuracy of our NDVI values for that target. We focus here on C40 cities, however this work could be expanded to global urban areas more broadly. These advances could help ensure policymakers have the tools and information needed to advocate for future natural space goals.

Our approach to translate C40's UND targets into NDVI terms makes it possible to estimate the health and subsequent economic benefits that could be achieved by meeting these targets. The use of open‐source, globally available data, allows cities around the world to track their progress and provides more context for the popular but not‐well understood NDVI metric. The specific conversions created in this work are made for the 96 C40 member cities, representing diverse cultural, political, and climatic contexts. Cities that were not included in this analysis but share similar climates and population sizes as cities in our study population, could use these estimates as a benchmark to which they could compare their own levels of urban natural space. The results of this study could provide useful information for municipal decision‐makers and provide leverage to increase political will for expanding urban natural space.

## Conflict of Interest

The authors declare no conflicts of interest relevant to this study.

## Supporting information

Supporting Information S1

## Data Availability

Data from the European Space Agency's (ESA) WorldCover and Sentinel‐2A datasets (Chander et al., 2009; Zanaga et al., 2021) were used to quantify urban natural space. All data are publicly available and accessed through Google Earth Engine (Gorelick et al., 2017). Data analysis and figure creation were done in Spyder 5.0 (Raybaut, 2009) and Stata 14.0 (StataCorp, 2015). The supplemental datasets referenced in Supporting Information S1 have been published in an open access repository (Martin, 2024a). All code used in this analysis is available in a Git repository (Martin, 2024b).
