# Supplementary material for: Tracking Progress Toward Urban Nature Targets Using Landcover and Vegetation Indices: A Global Study for the 96 C40 Cities
Source: Geohealth. 2024 Feb 26;8(3):e2023GH000996. doi: 10.1029/2023GH000996 (PMC10897363; doi:10.1029/2023GH000996)
Supplement: Supplementary file 1 — Supporting Information S1 [file GH2-8-e2023GH000996-s001.docx]

*GeoHealth*

Supporting Information for

**Tracking progress towards urban nature targets using landcover and vegetation indices: A global study for the 96 C40 Cities**

Greta K. Martin^1^, Katelyn O’Dell^1^, Patrick L. Kinney^2^, Marcia Pescador Jimenez^2^, David Rojas-Rueda, Robert Canales^1^, Susan C. Anenberg^1^

^1^The George Washington University Milken Institute of Public Health, Washington, DC

^2^Boston University School of Public Health, Boston, MA

^3^Colorado School of Public Health, Aurora, CO

**Contents of this file**

Data Set S1 to S2

Figures S1 to S23

**Introduction**

The supporting information includes two datasets in .csv format published in an open-access data repository here: <https://doi.org/10.5281/zenodo.10569694>. The first is for the main analysis using Urban Centre Database bounds and includes city-level summary measures. These include measures of natural space and population as well as model diagnostics and outputs. The second is a parallel dataset of the sensitivity analysis using the self-defined C40 urban bounds.

In addition, there are 23 supplemental figures included that provide more in-depth results than those provided in the main text as well as some results from sensitivity analyses.

One city, Jakarta, Indonesia, was missing age- and sex-specific gridded population data. For this city, we use WorldPop gridded total population data multiplied by the proportion of the Indonesian population that is 20 years or older. The 2020 Indonesian population was used to estimate the population pyramid of Jakarta (World Population Prospects 2020, 2022).

**Supplemental Data Set 1.** City-level natural space and population measures using Urban Centre Database (UCDB) urban bounds. Model diagnostics and outputs are also included.

**Supplemental Data Set 2.** City-level natural space and population measures using C40 self-defined urban bounds. Model diagnostics and outputs are also included.


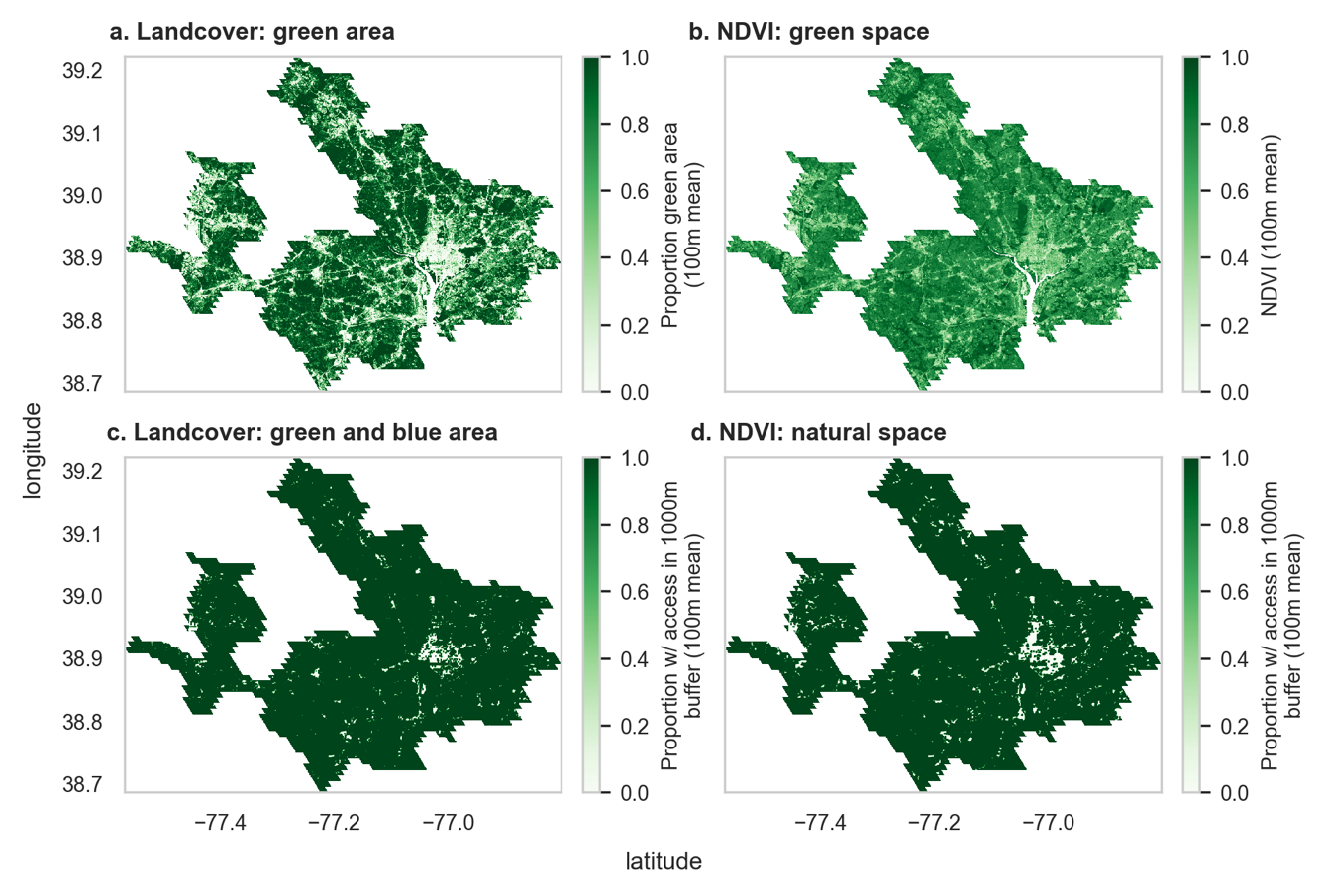


Figure S1. Natural space regression inputs for an example city, Washington, DC, for 2020. Panels a and b show the metrics used to assess the Quality Total Cover target while panels c and d show the metrics used to assess the Equitable Spatial Distribution target. Panel a is the proportion of green area in each 100m pixel, panel b the mean 100m normalized difference vegetation index (NDVI), panel c the proportion of the 100m pixel with access to landcover-based natural space within a 1000m buffer, and panel D the proportion of the 100m pixel with access to NDVI-based natural space within a 1000m buffer (using a threshold of the predicted NDVI value where proportion of green area=0.75).

***
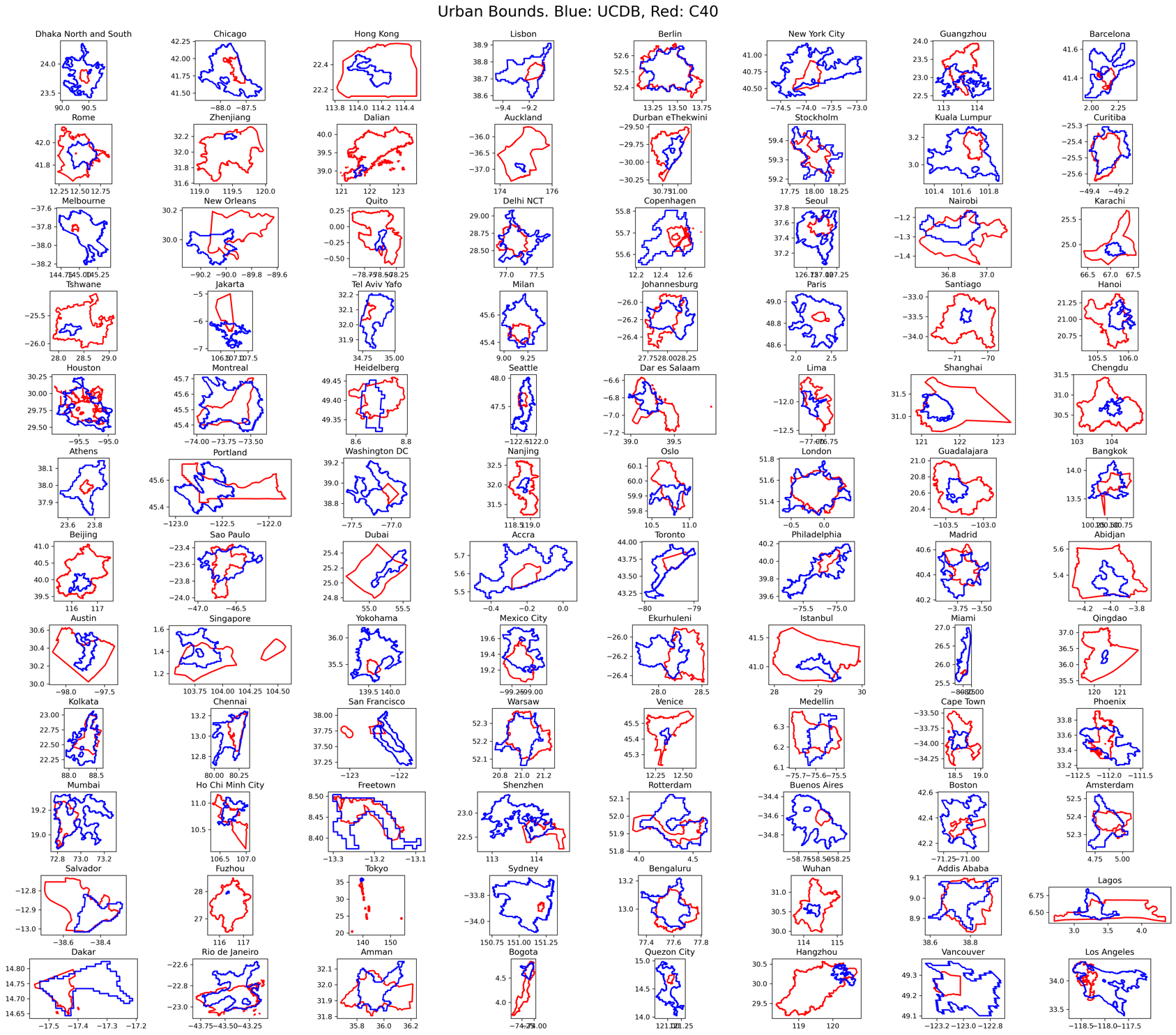
***

***Figure S2.*** *Comparison of Global Human Settlement Urban City Database (GHS-UCDB) urban bounds (shown in blue) and C40 self-defined city definitions (shown in red). We have used the GHS-UCDB bounds for our primary analysis as they are constructed in a consistent manner using information on population density and built-up area. However, we conducted a sensitivity analysis using the urban definitions provided by C40 cities. These self-defined bounds tend to represent a smaller area than those of the GHS-UCDB, though this is not always the case, particularly in Chinese C40 cities. The C40 defined shapes are shown in red and the GHS-UCDB bounds in blue.*


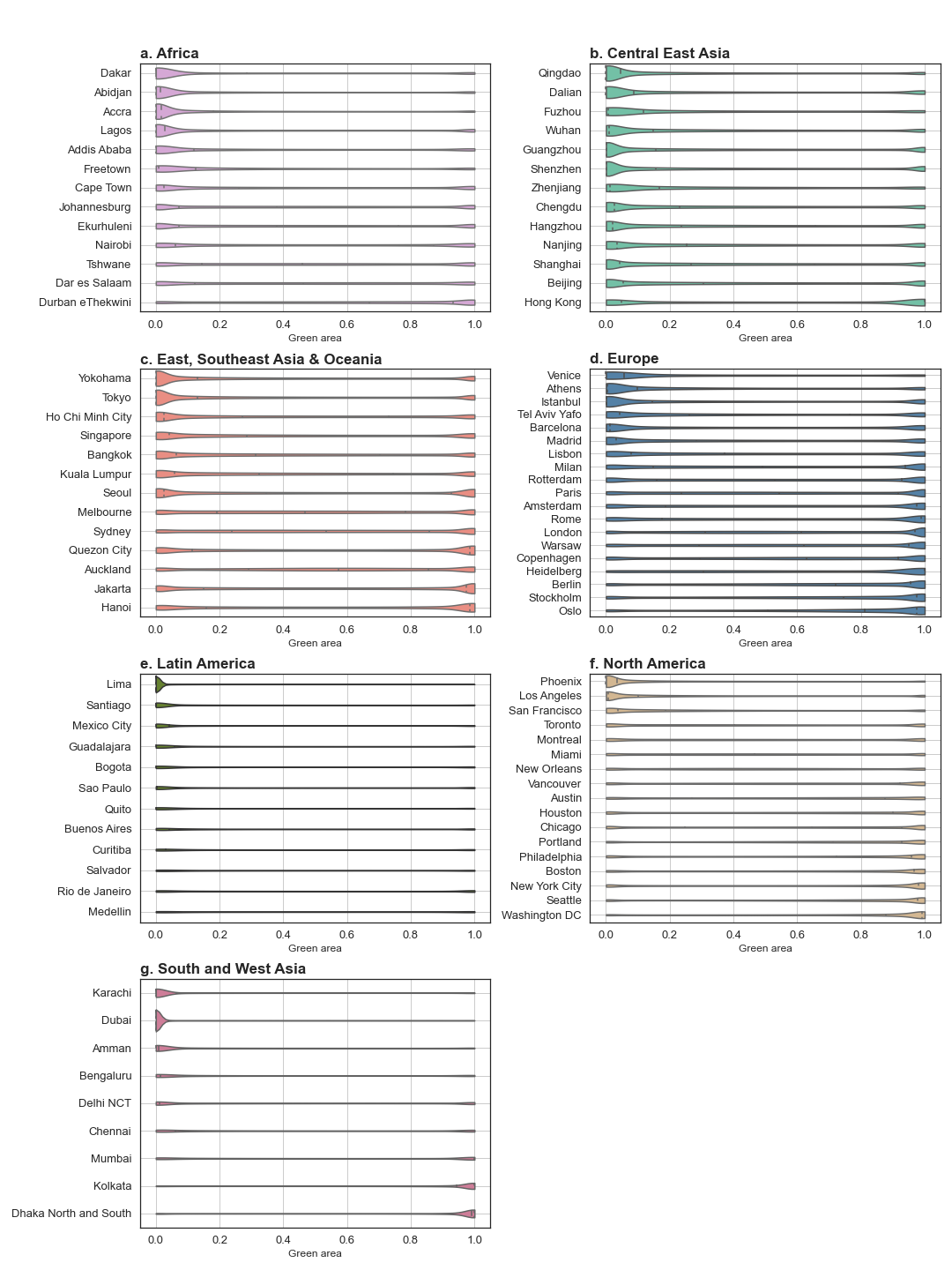
Figure S3. Distribution of 100m proportion of green area for each pixel in C40 cities within each world region. Quartiles of green area are shown by vertical lines. These distributions do not include blue space.


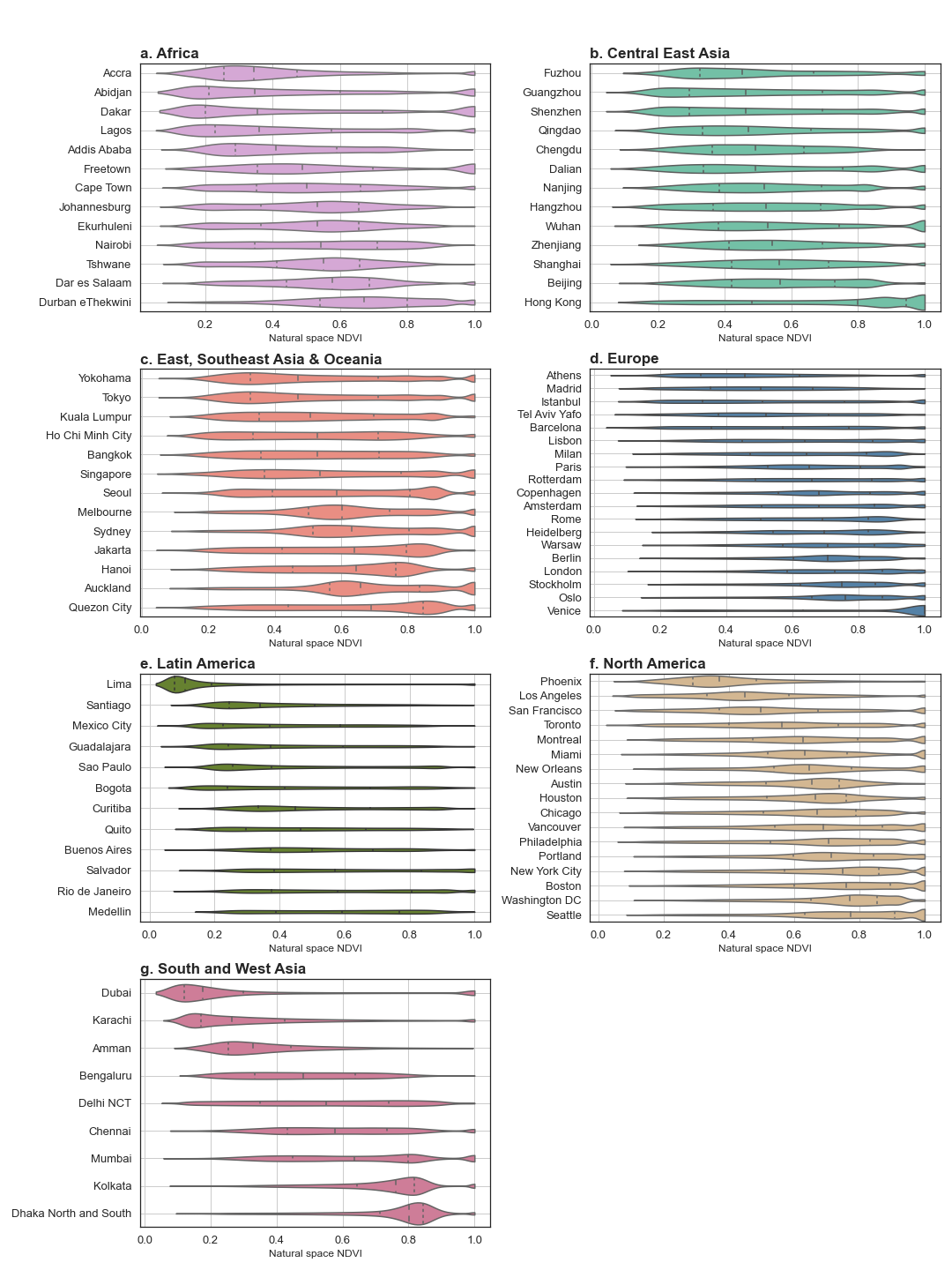
Figure S4. Distribution of natural space NDVI 100m values for each pixel in C40 cities within each world region. Quartiles of natural space NDVI are shown by vertical lines.


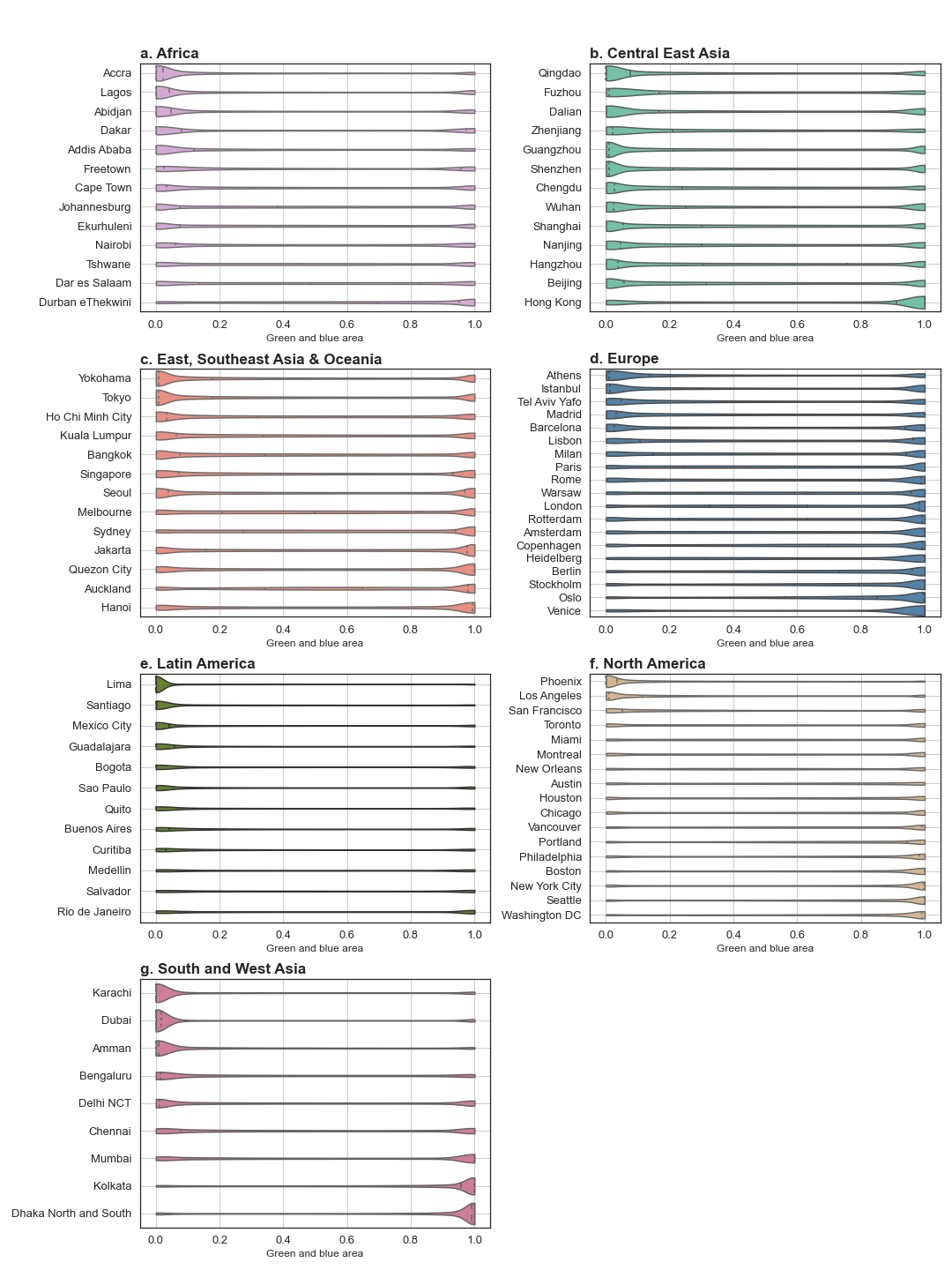
Figure S5. Distribution of 100m proportion of green and blue area for each pixel in C40 cities within each world region. Quartiles of green and blue area are shown by vertical lines.

**
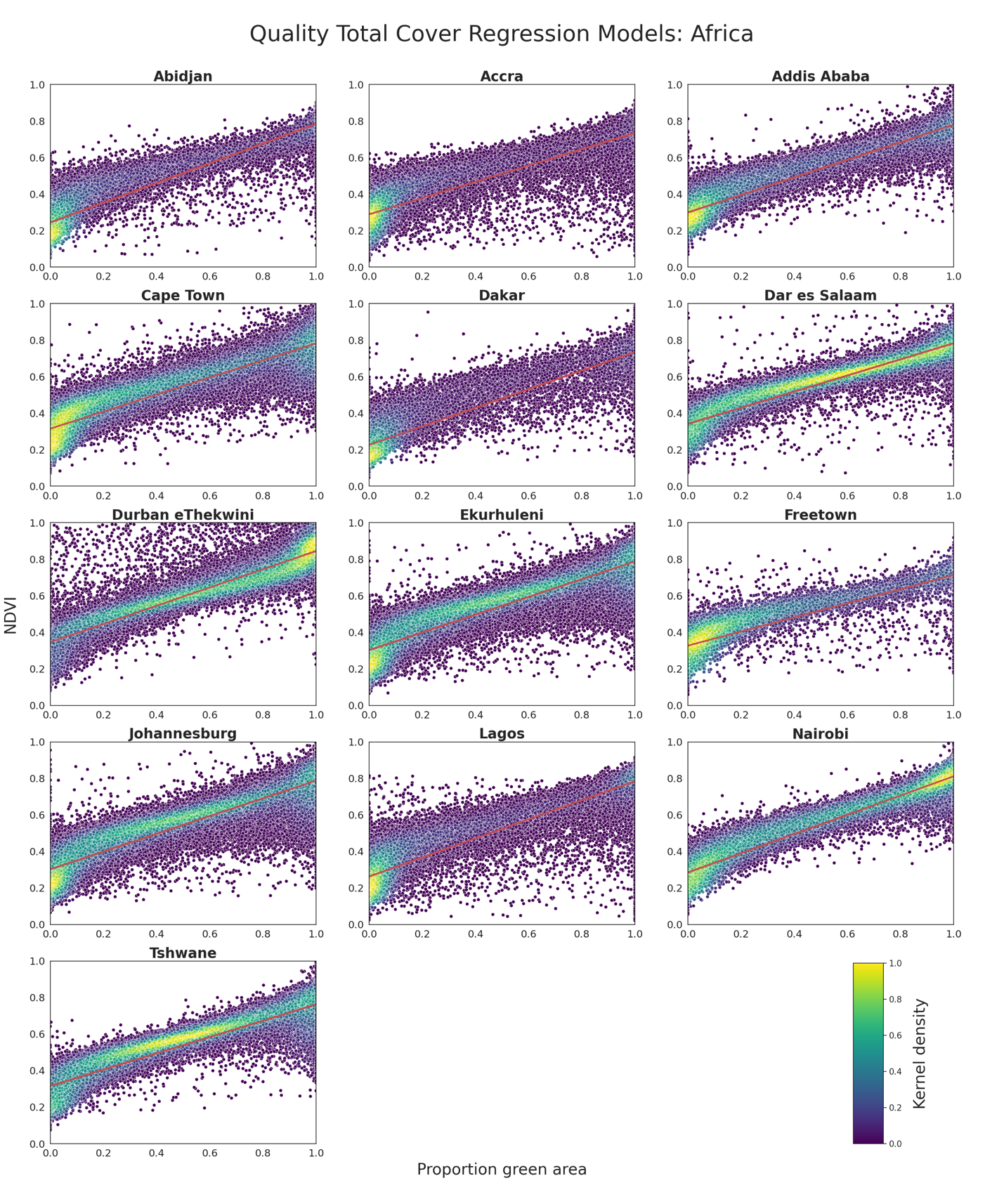
**

***Figure S6.*** *Kernel density scatter plots of the mean NDVI and mean proportion green area for each 100m pixel in each city in the region of Africa. The light-yellow shade shows higher values of the probability density function. The ordinary least squares (OLS) regression line is overlaid in red.*

*
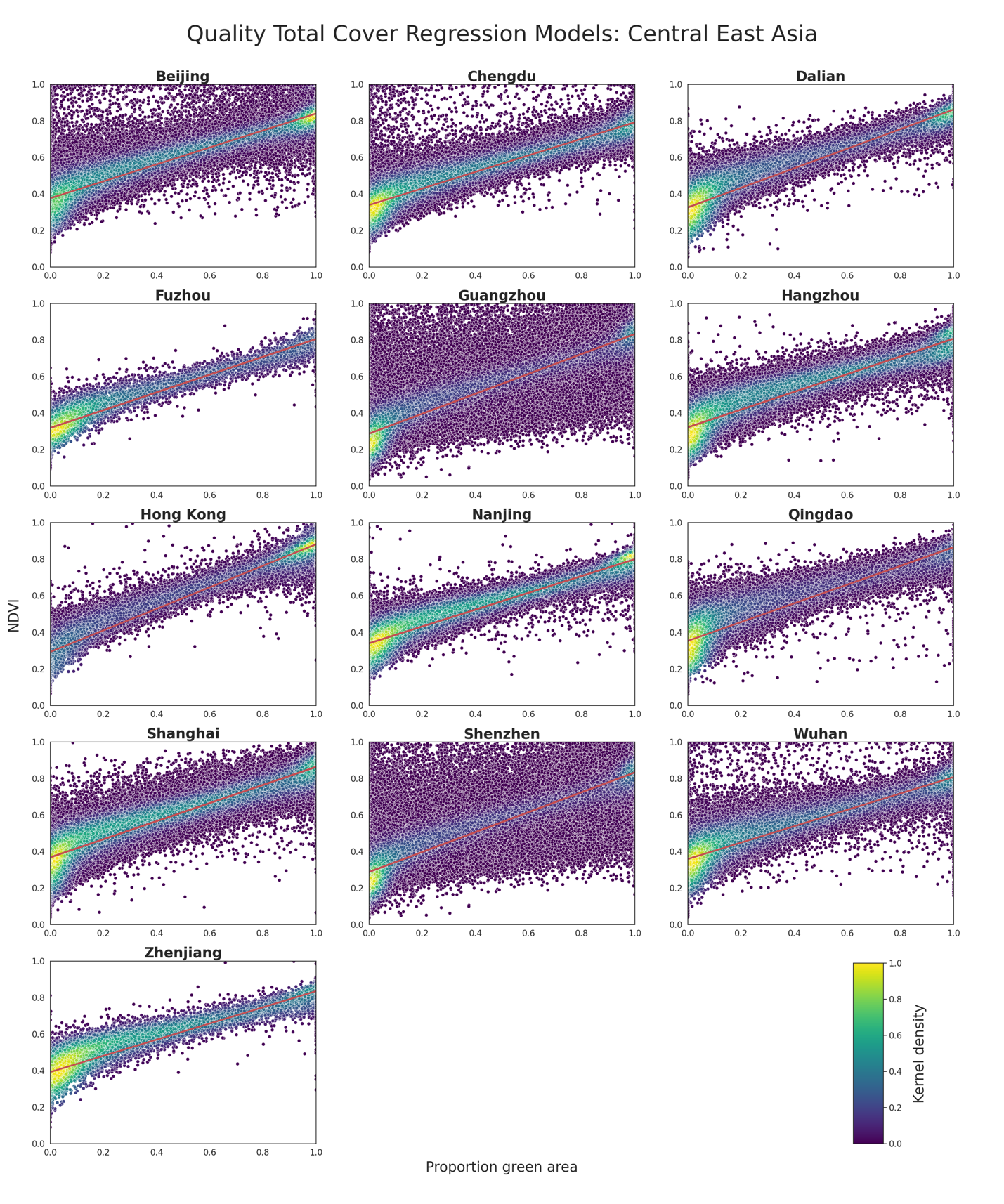
****Figure S7.*** *The same plot as S6 but for the region of Central East Asia.*

***
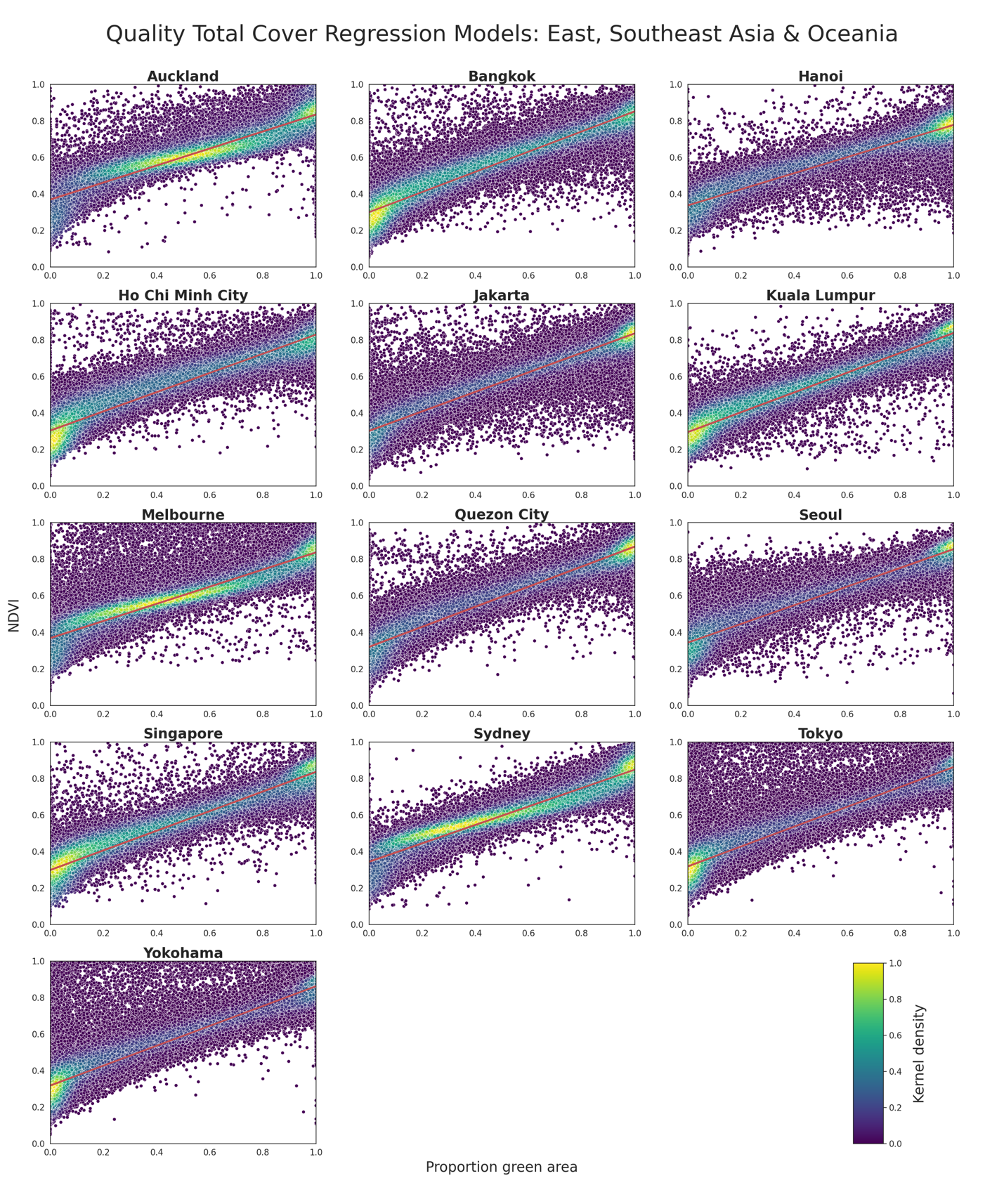
***

***Figure S8.*** *The same plot as S6 but for the region of East, Southeast Asia, and Oceania.*


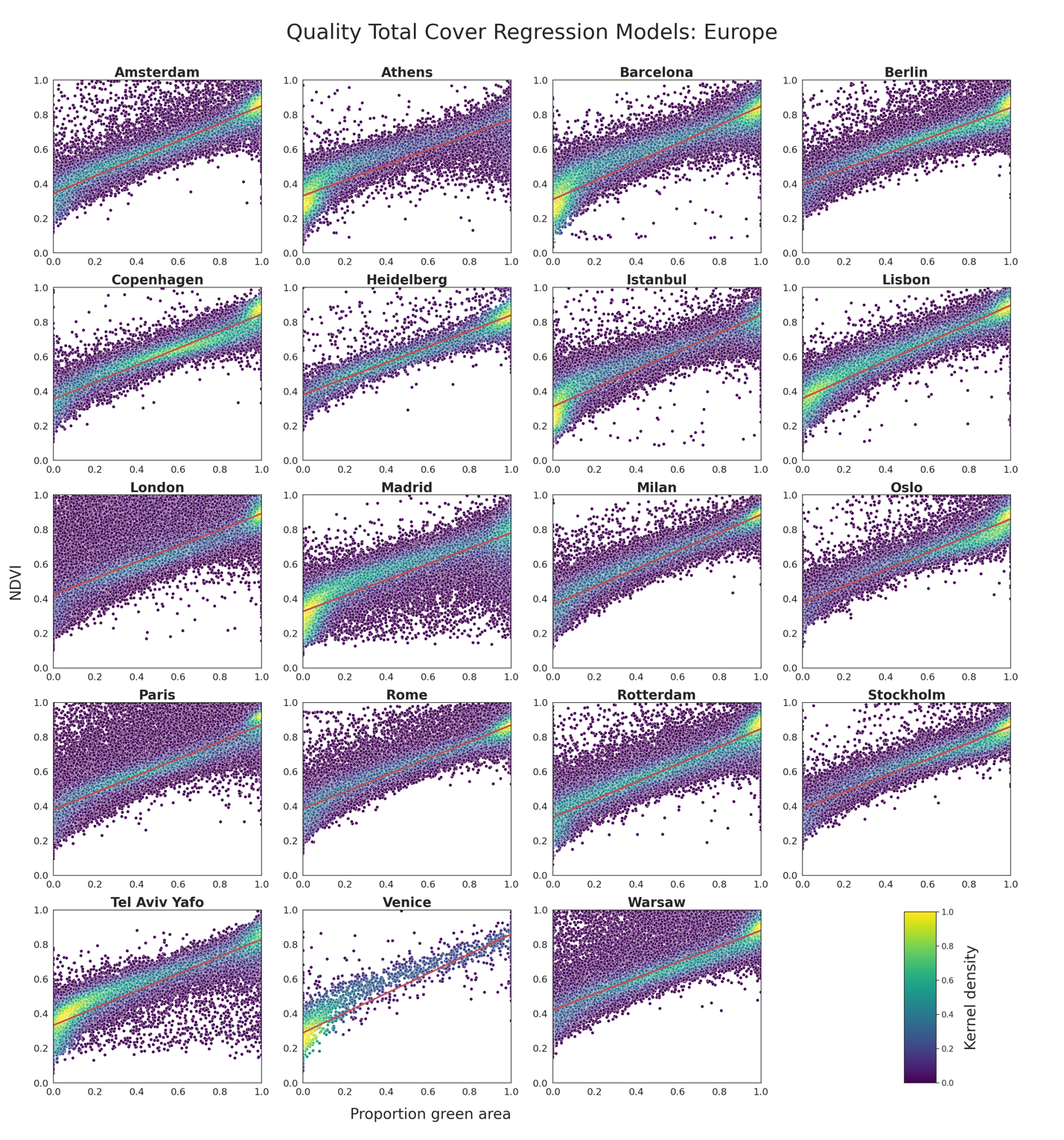
***Figure S9.*** *The same plot as S6 but for the region of Europe.*

***
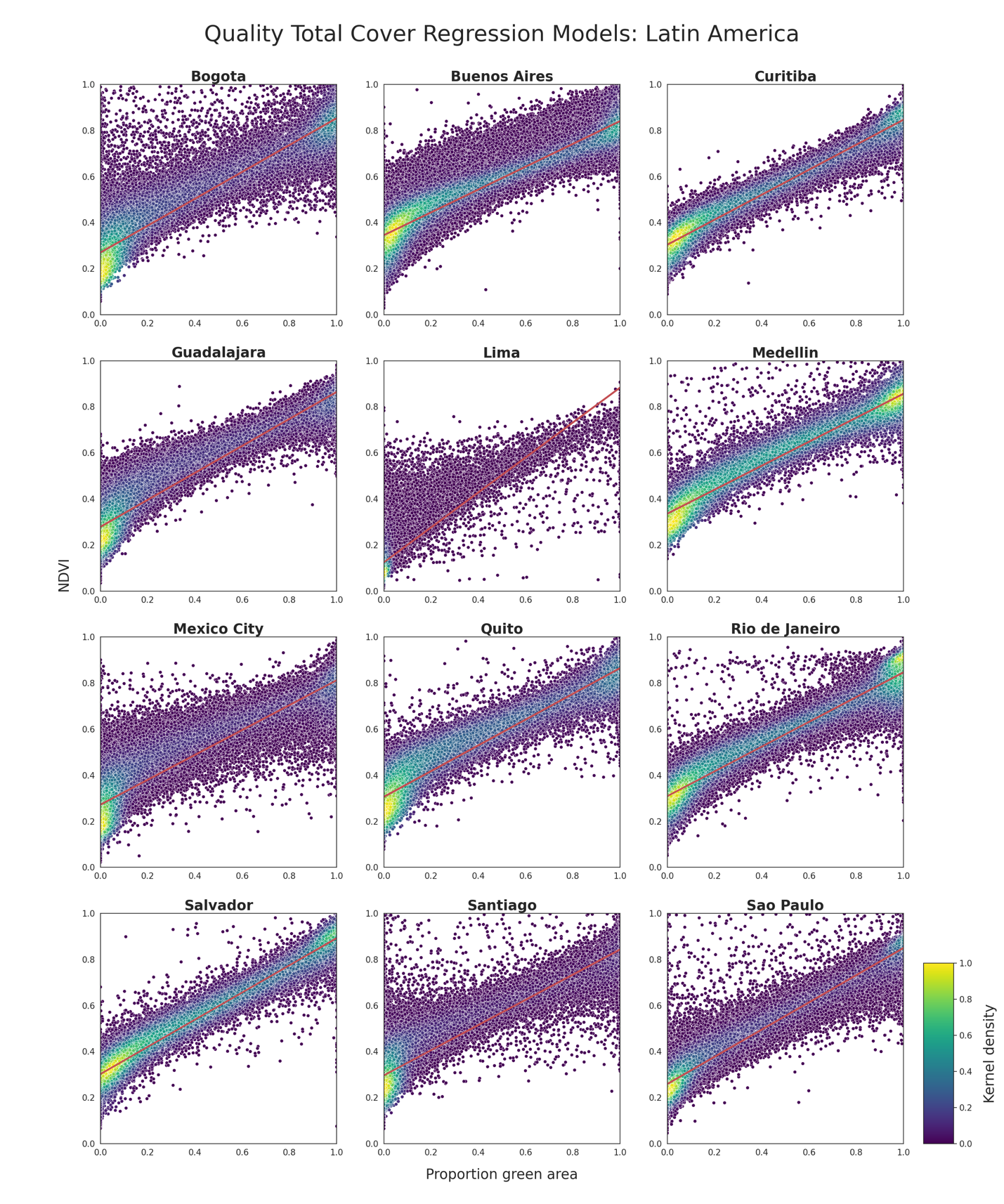
***

***Figure S10.*** *The same plot as S6 but for the region of Latin America.*

*
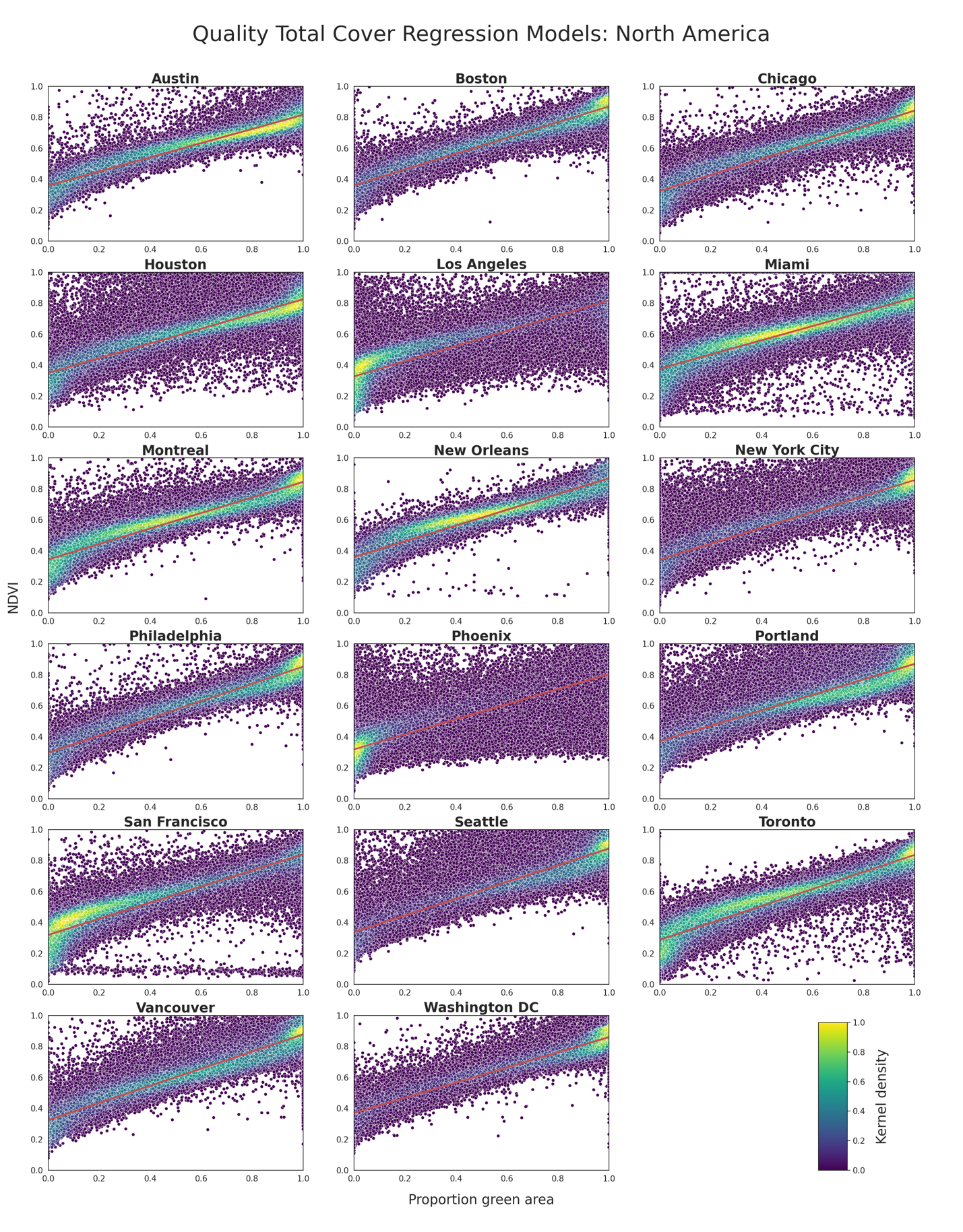
*

***Figure S11.*** *The same plot as S6 but for the region of North America.*

*
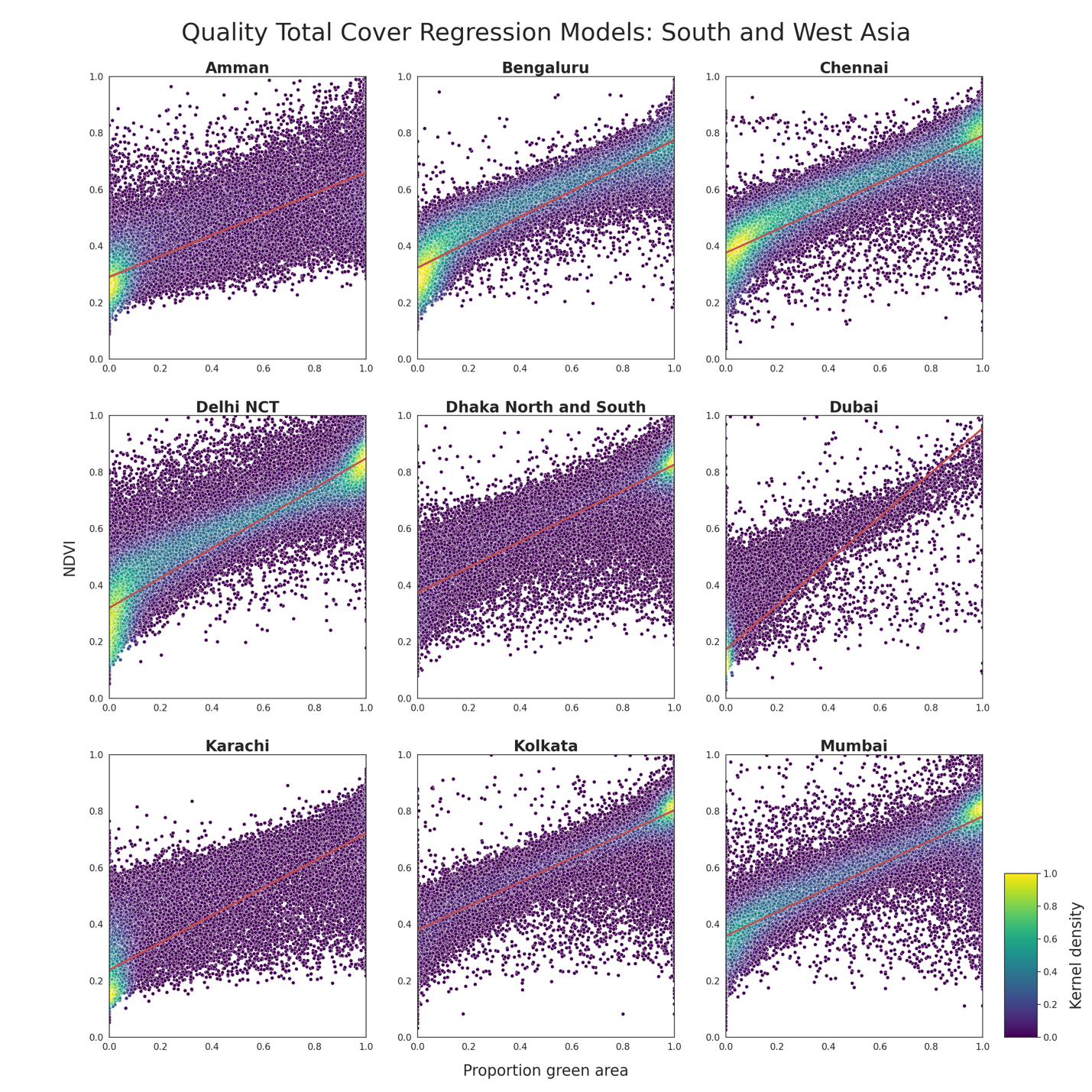
*

***Figure S12.*** *The same plot as S6 but for the region of South and West Asia.*


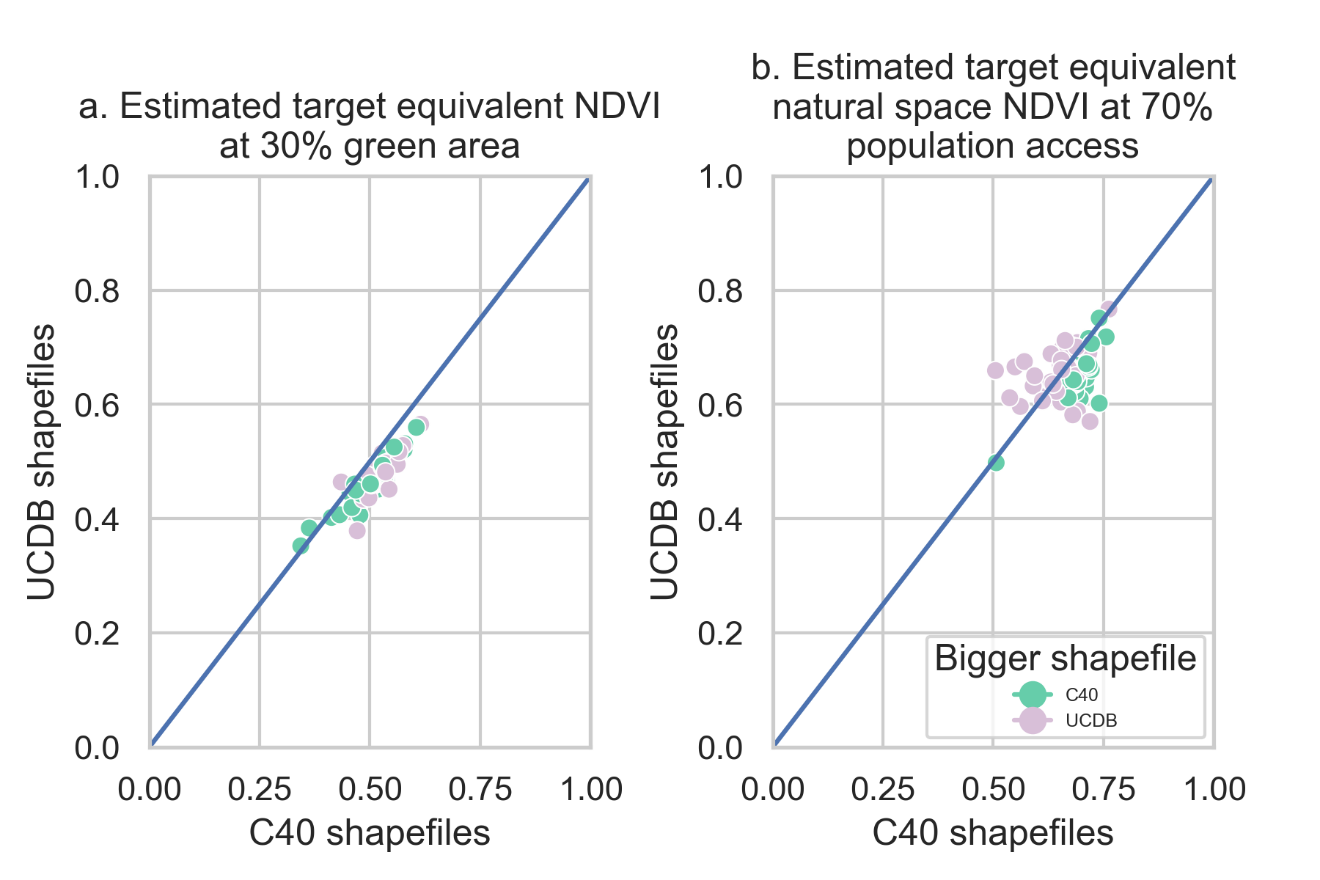


**Figure S13.** Comparison of estimated Urban Nature Declaration target-equivalent NDVI-scale values by urban boundary definition. Panel a shows the estimated NDVI value equivalent of achieving the Quality Total Cover target, or 30% green area. Panel b shows the estimated natural space NDVI value equivalent of achieving the Equitable Spatial Distribution target, or 70% area with access to natural space. Each dot represents a city, with purple dots representing cities in which the Global Human Settlement Urban Centres Database (UCDB) urban definition is a larger area and green dots showing cities for which the C40 urban definition is larger.


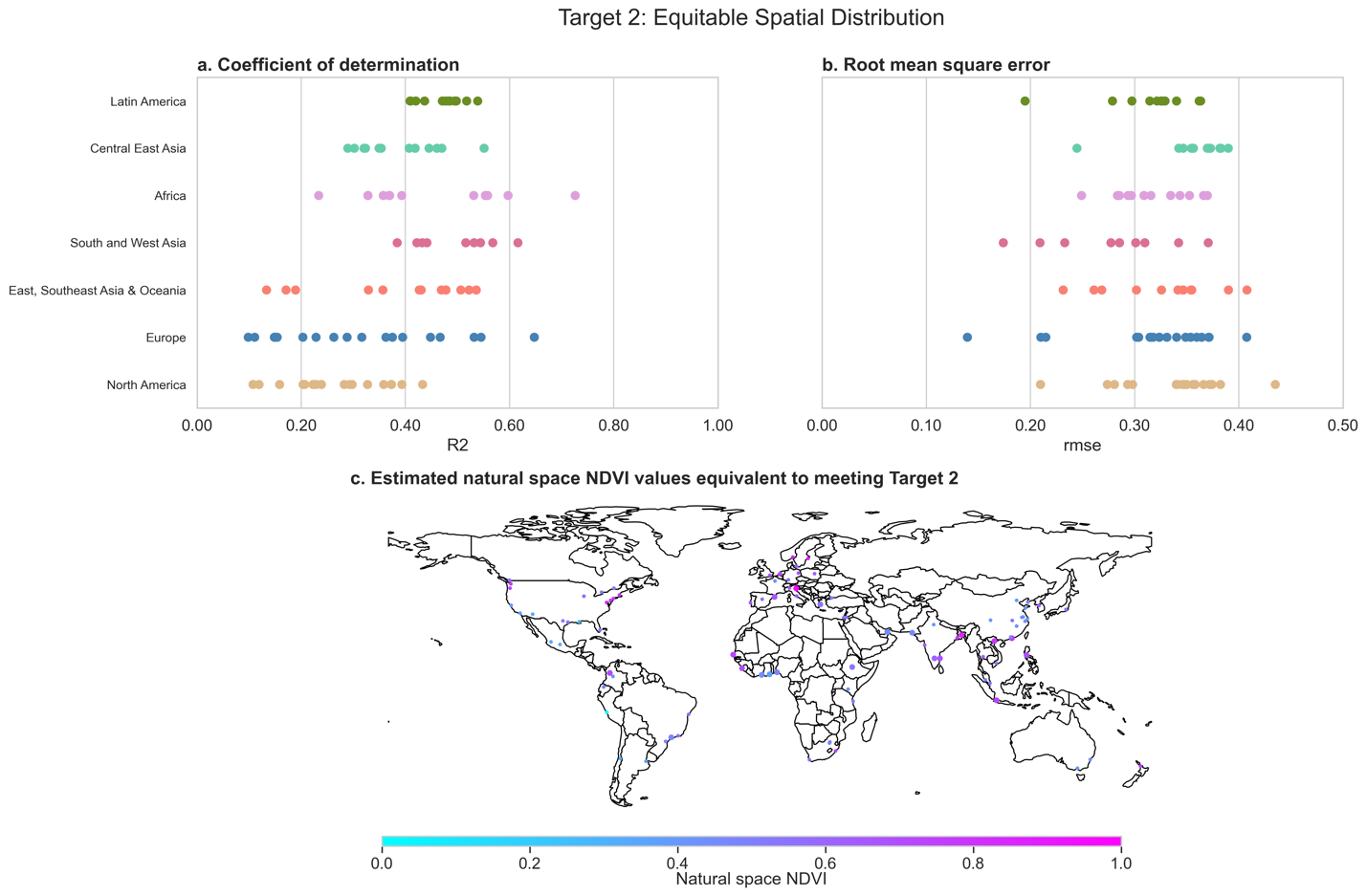


***Figure S14.*** *Equivalent to Figure 5 in the main text, but using* *the predicted NDVI value at 0.90 proportion of green area as the threshold for identifying greenspace. Each dot represents a city. Panels a and b show the model fit statistics by region. Panel a shows the adjusted R^2^ value and panel b shows the root mean square error (rmse). Panel c shows the predicted natural space NDVI value where 0.70 of the area, and thus population, has access to sufficient nearby natural space. Models with poor fit (R^2^ less than 0.50) are shown with smaller dots.*

***
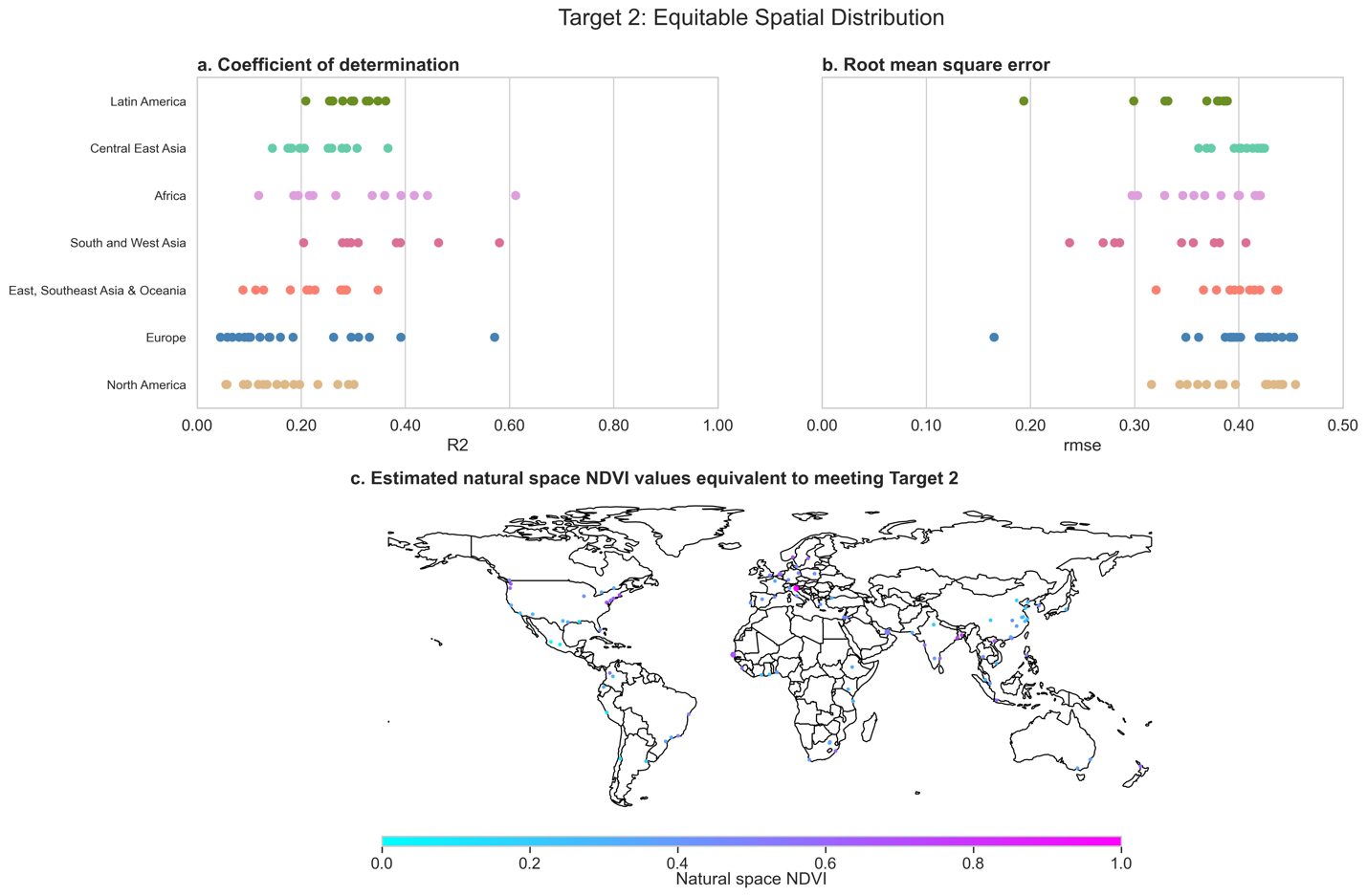
Figure S15.*** *Equivalent to Figure 5 in the main text, but using* *the predicted NDVI value at 1.0 proportion of green area as the threshold for identifying greenspace. Each dot represents a city. Panels a and b show the model fit statistics by region. Panel a shows the adjusted R^2^ value while panel b shows the root mean square error (rmse). Panel c shows the predicted natural space NDVI value where 0.70 of the area, and thus population, has access to sufficient nearby natural space. Models with poor fit (R^2^ less than 0.50) are shown with small dots.*

***
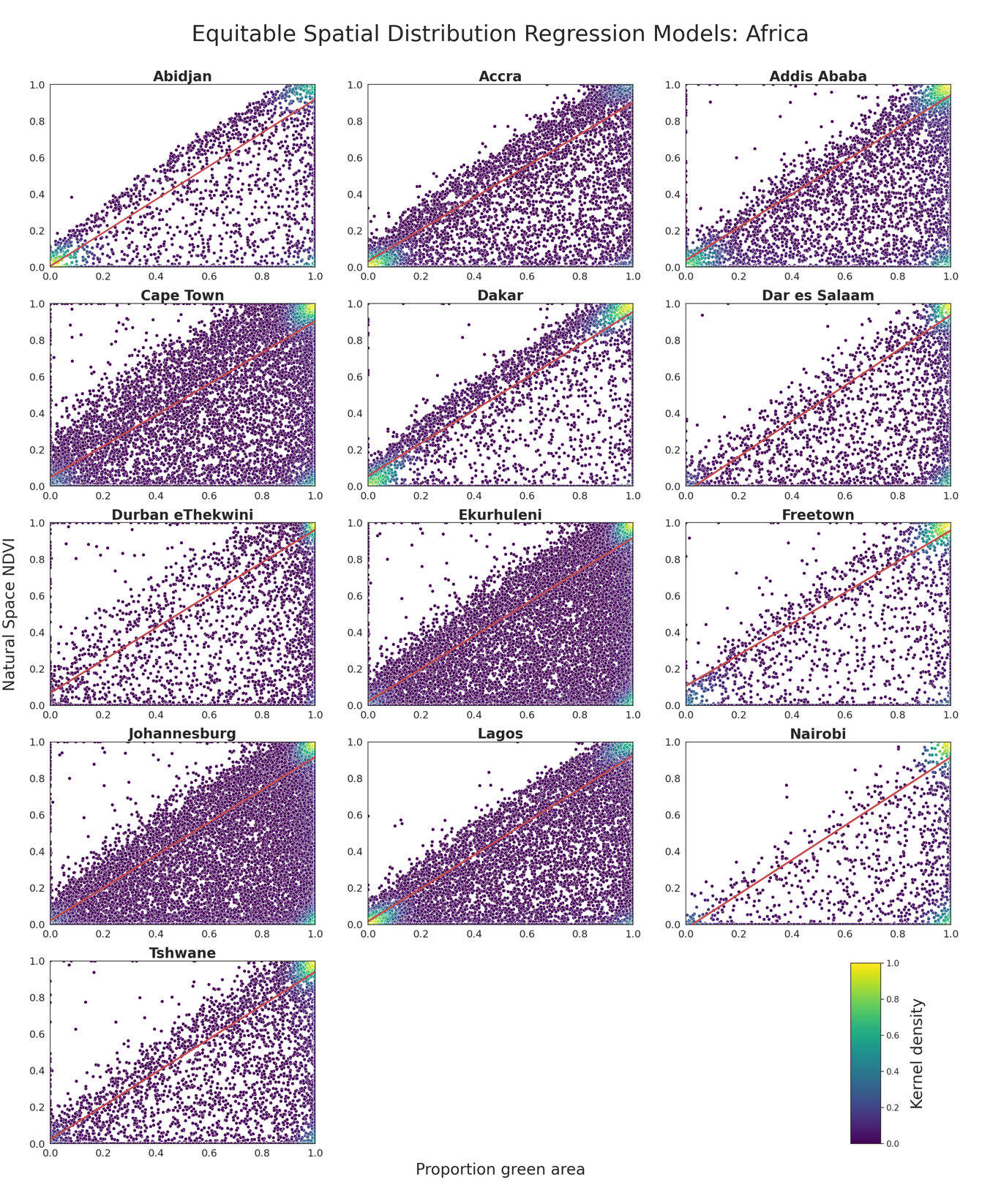
***

***Figure S16.*** *Kernel density scatter plots of the proportion of each 100m pixel with access to a minimum of 0.5 hectares of natural space (as defined by meeting a minimum NDVI threshold or higher) within a 1000m buffer and the proportion of the 100m grid cell with natural space (as defined by the landcover dataset) within a 1000m buffer in each city in the region of Africa. The light-yellow shade shows higher values of the probability density function. The ordinary least squares (OLS) regression line is overlaid in red.*

*
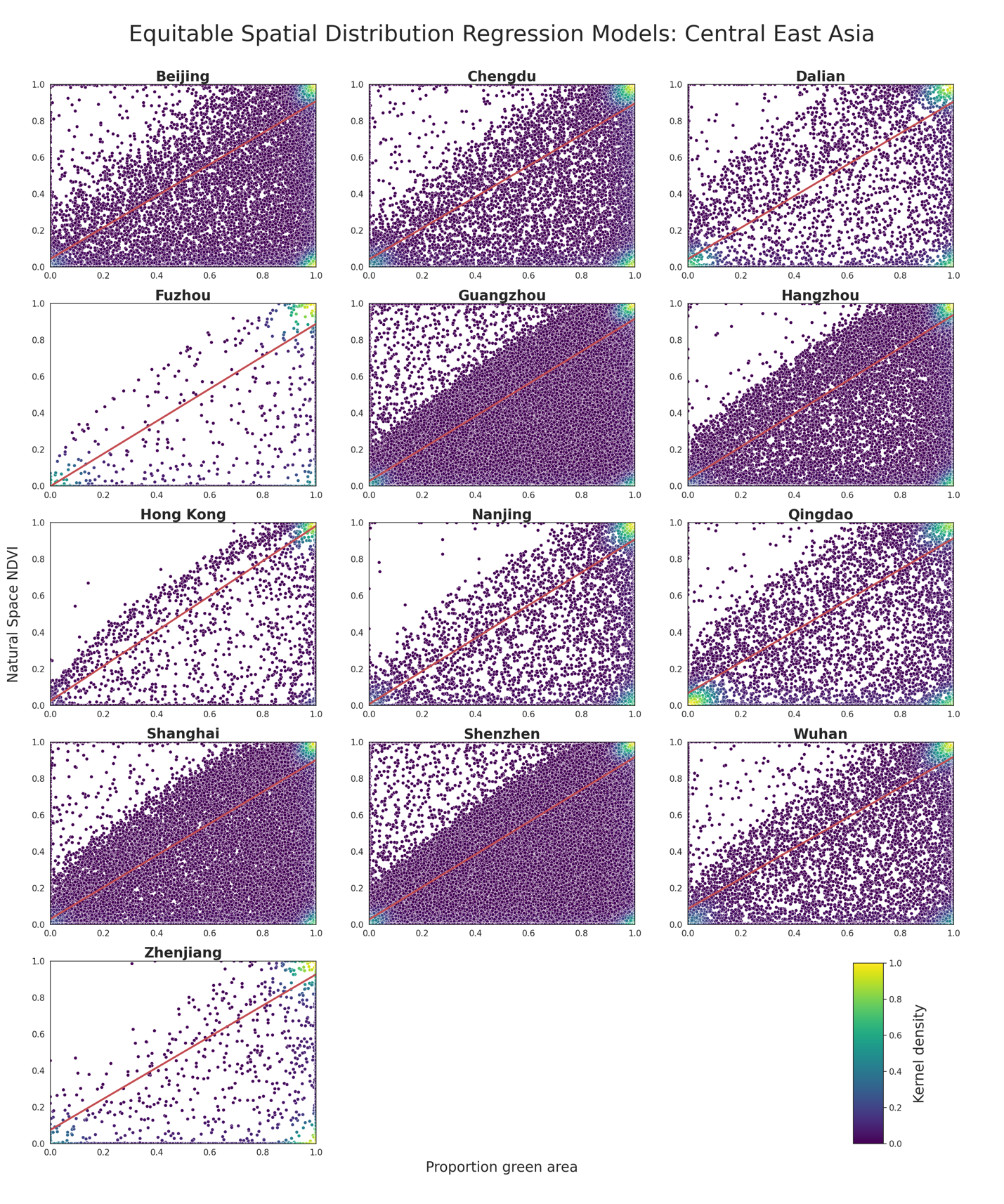
*

***Figure S17.*** *The same plot as S16 but for the region of Central East Asia.*


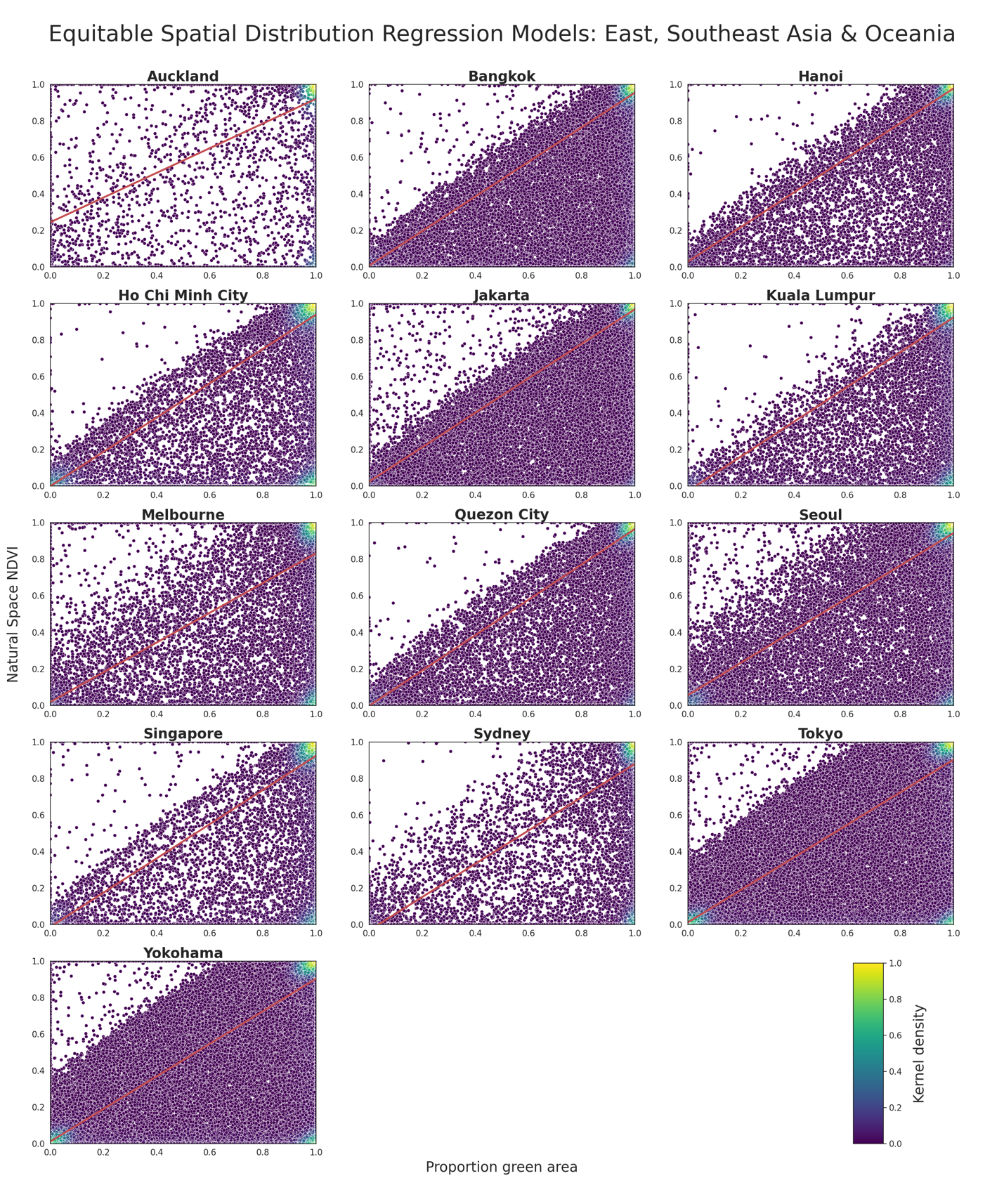


***Figure S18.*** *The same plot as S16 but for the region of East, Southeast Asia, and Oceania.*

***
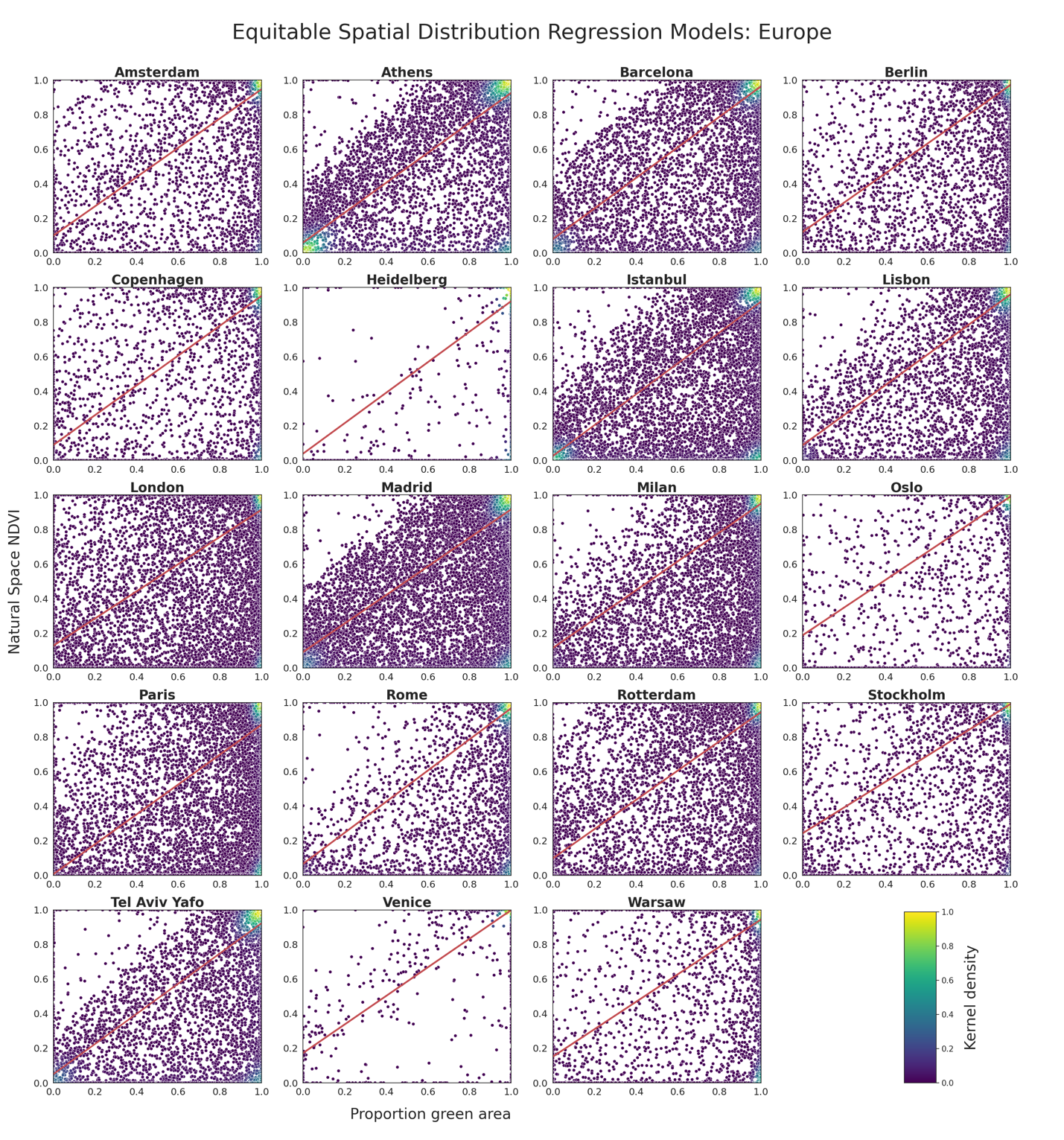
***

***Figure S19.*** *The same plot as S16 but for the region of Europe.*


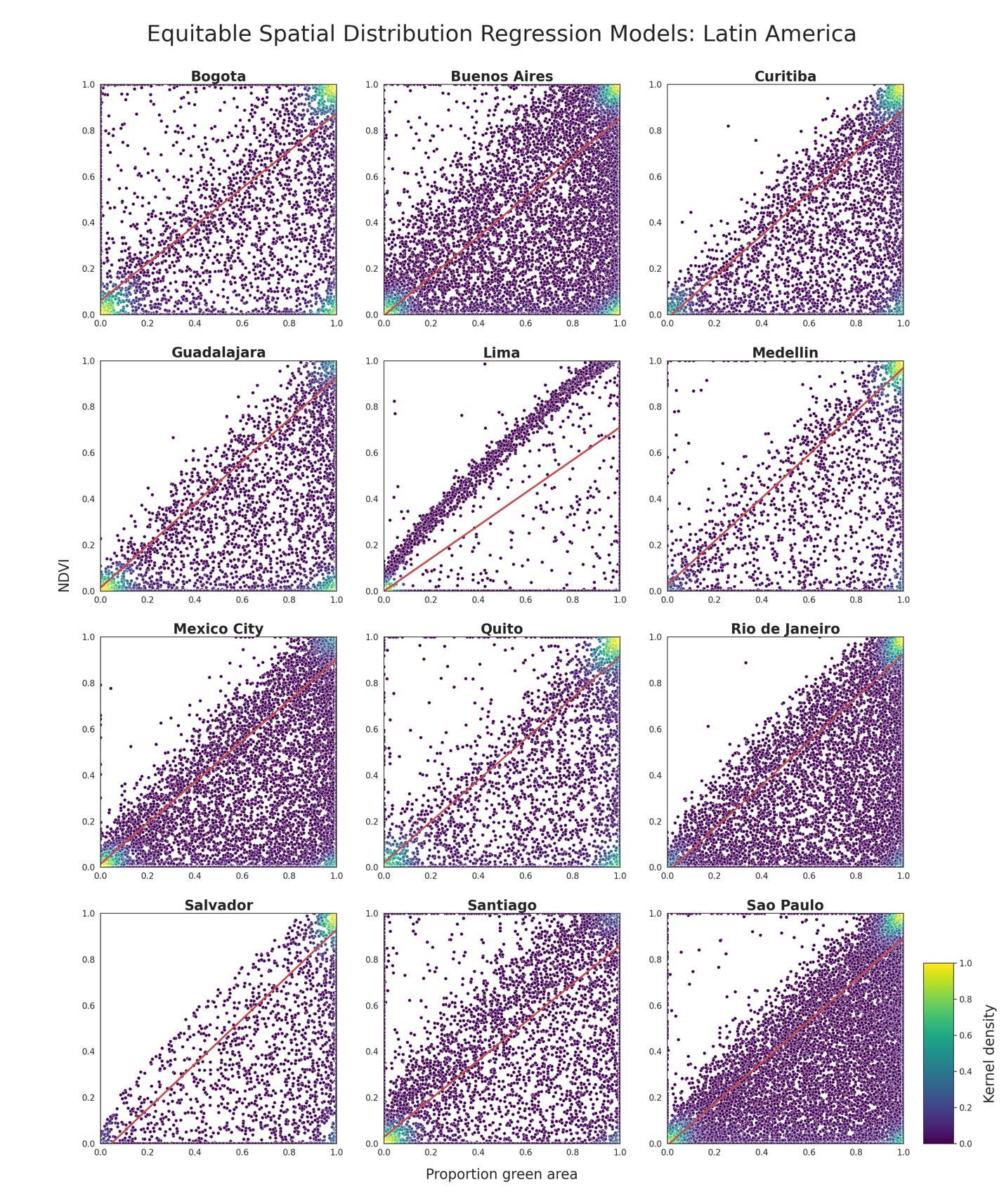


***Figure S20.*** *The same plot as S16 but for the region of Latin America.*


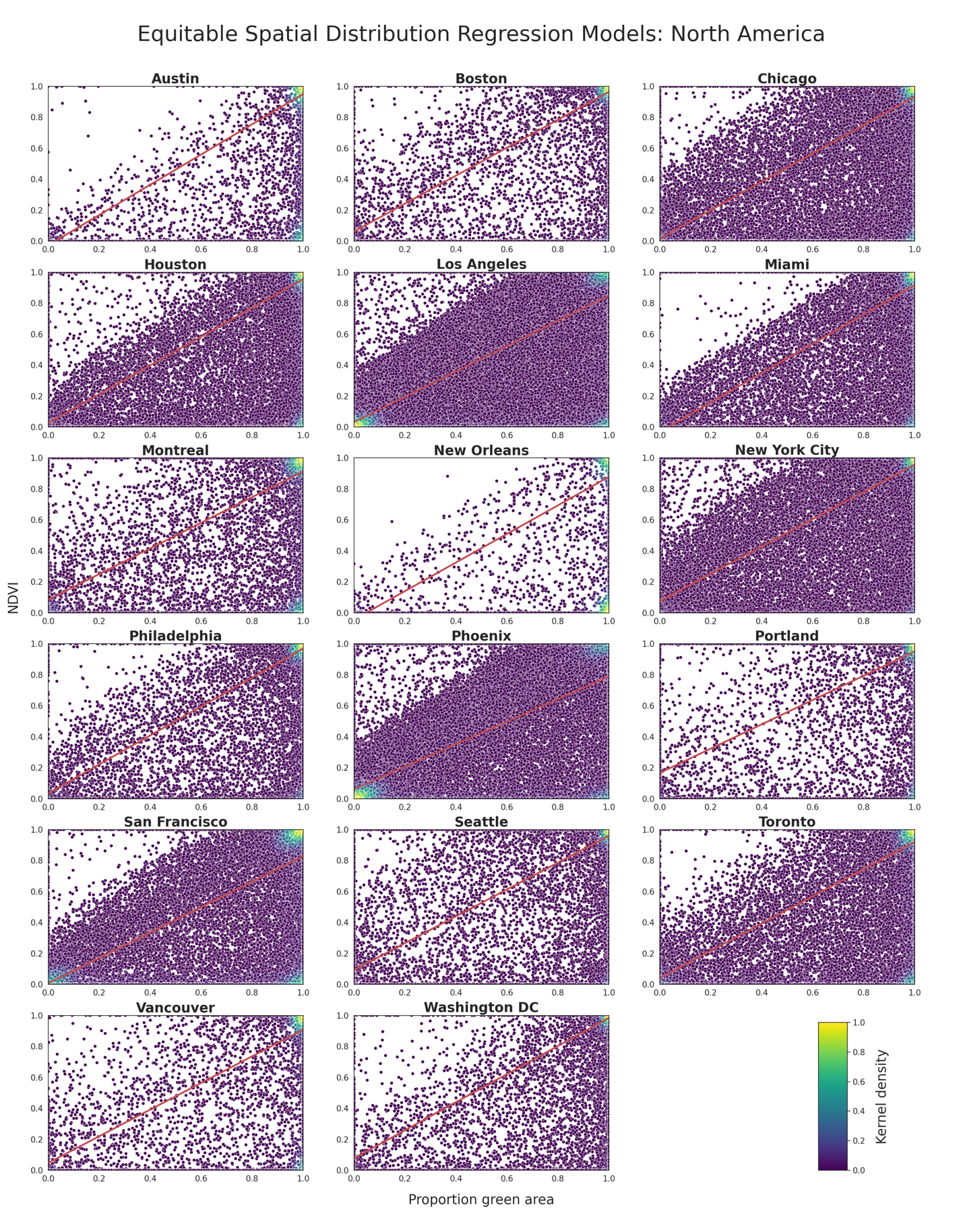


***Figure S21.*** *The same plot as S16 but for the region of North America.*

*
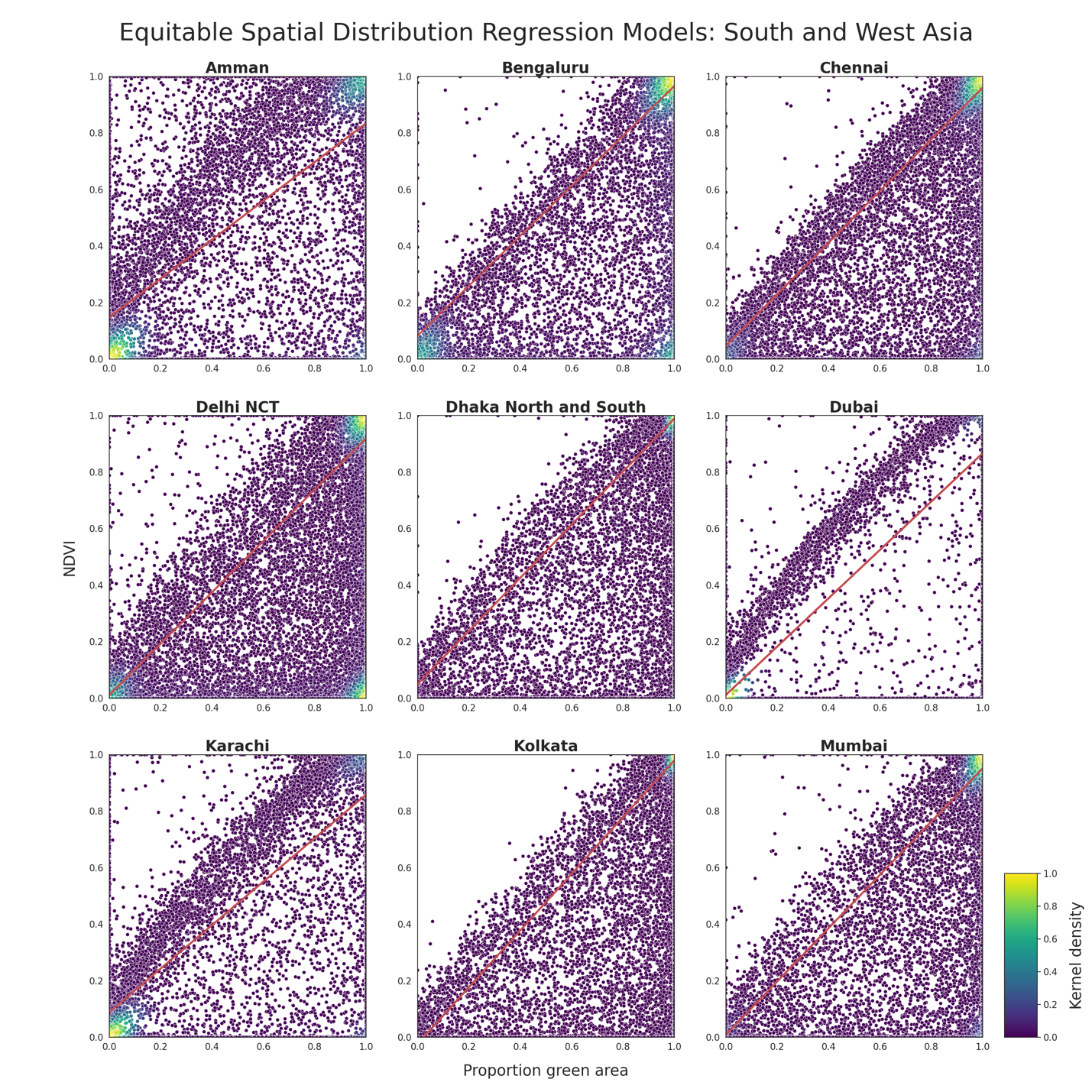
*

***Figure S22.*** *The same plot as S16 but for the region of South and West Asia.*

We assessed how our estimates of the Equitable Spatial Distribution target aligned with Trust for Public Land’s ParkServe® data (Trust for Public land, 2024), which are available for 15,000 U.S. cities and towns and include a measure of the proportion of the population with access to publicly available greenspace with a 10 minute or half mile (~800m) walk. There are some notable differences between these two measures. First, we use the Urban Centres Database while Parkserve uses the U.S. Census Urban Area designations to define urban bounds. Second, we use a satellite-derived measure of natural space that is inclusive of both green and blue space of at least half a hectare while Parkserve uses data collected from each city on publicly accessible parks, trails and open space. Third, we use a 1000m buffer to approximate access within a 15 minute walk while Parkserve uses Esri’s Street Map Premium network dataset which takes into account walking barriers. Lastly, our data is from 2020, while to the best of our knowledge the data on Parkserve’s mapping tool is from 2022.

Given the discrepancies in methodologies, we expected our measure of access to be higher. This was the case for 11 of the 14 U.S. cities. In three cities (Los Angeles, New York, and San Francisco), the opposite relationship was observed. As these are the three largest American C40 cities, it is possible that the difference in urban boundary definition is having a larger effect in these cases.

*
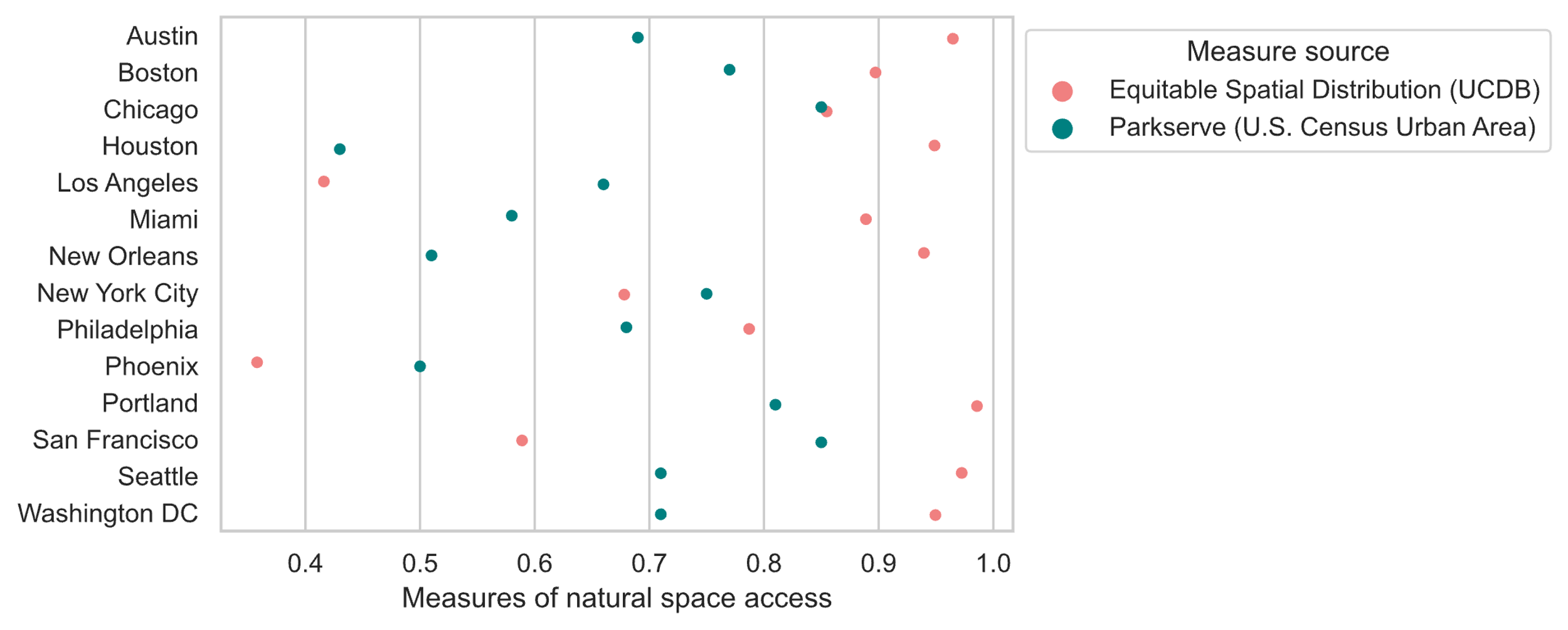
*

***Figure S23.*** *A comparison of the proportion of the population with access to at least 0.5 hectares of green or blue space within a 15 minute or 1000m walk used in this paper to assess the Equitable Spatial Distribution target and Trust for Public Land’s Parkserve measure of the proportion of the population with access to publicly available greenspace with a 10 minute or half mile walk (~805m). The Parkserve measure is depicted in teal, while the metric developed in this paper is pink.*
